# IFITM Proteins Restrict Viral Membrane Hemifusion

**DOI:** 10.1371/journal.ppat.1003124

**Published:** 2013-01-24

**Authors:** Kun Li, Ruben M. Markosyan, Yi-Min Zheng, Ottavia Golfetto, Brittani Bungart, Minghua Li, Shilei Ding, Yuxian He, Chen Liang, James C. Lee, Enrico Gratton, Fredric S. Cohen, Shan-Lu Liu

**Affiliations:** 1 Department of Molecular Microbiology and Immunology, Bond Life Sciences Center, University of Missouri, Columbia, Missouri, United States of America; 2 Department of Molecular Biophysics and Physiology, Rush University Medical Center, Chicago, Illinois, United States of America; 3 Laboratory for Fluorescence Dynamics, Biomedical Engineering Department, University of California, Irvine, Irvine, California, United States of America; 4 Department of Biological Engineering, University of Missouri, Columbia, Missouri, United States of America; 5 Department of Microbiology and Immunology, McGill University, Montreal, Quebec, Canada; 6 MOH Key Laboratory of Systems Biology of Pathogens, Institute of Pathogen Biology, Chinese Academy of Medical Sciences & Peking Union Medical College, Beijing, China; 7 McGill AIDS Centre, Lady Davis Institute, Montreal, Quebec, Canada; Fred Hutchinson Cancer Research Center, United States of America

## Abstract

The interferon-inducible transmembrane (IFITM) protein family represents a new class of cellular restriction factors that block early stages of viral replication; the underlying mechanism is currently not known. Here we provide evidence that IFITM proteins restrict membrane fusion induced by representatives of all three classes of viral membrane fusion proteins. IFITM1 profoundly suppressed syncytia formation and cell-cell fusion induced by almost all viral fusion proteins examined; IFITM2 and IFITM3 also strongly inhibited their fusion, with efficiency somewhat dependent on cell types. Furthermore, treatment of cells with IFN also markedly inhibited viral membrane fusion and entry. By using the Jaagsiekte sheep retrovirus envelope and influenza A virus hemagglutinin as models for study, we showed that IFITM-mediated restriction on membrane fusion is not at the steps of receptor- and/or low pH-mediated triggering; instead, the creation of hemifusion was essentially blocked by IFITMs. Chlorpromazine (CPZ), a chemical known to promote the transition from hemifusion to full fusion, was unable to rescue the IFITM-mediated restriction on fusion. In contrast, oleic acid (OA), a lipid analog that generates negative spontaneous curvature and thereby promotes hemifusion, virtually overcame the restriction. To explore the possible effect of IFITM proteins on membrane molecular order and fluidity, we performed fluorescence labeling with Laurdan, in conjunction with two-photon laser scanning and fluorescence-lifetime imaging microscopy (FLIM). We observed that the generalized polarizations (GPs) and fluorescence lifetimes of cell membranes expressing IFITM proteins were greatly enhanced, indicating higher molecularly ordered and less fluidized membranes. Collectively, our data demonstrated that IFITM proteins suppress viral membrane fusion before the creation of hemifusion, and suggested that they may do so by reducing membrane fluidity and conferring a positive spontaneous curvature in the outer leaflets of cell membranes. Our study provides novel insight into the understanding of how IFITM protein family restricts viral membrane fusion and infection.

## Author Summary

Many pathogenic viruses contain an envelope that must fuse with the cell membrane in order to gain entry and initiate infection. This process is mediated by one or more glycoproteins present on the surface of the virions, known as viral fusion proteins. Recently, a family of interferon-inducible transmembrane (IFITM) protein has been shown to block viral infection, including those of highly pathogenic viruses. Here we provide evidence that these IFITM proteins potently suppress membrane fusion induced by representatives of all three classes of viral fusion proteins. Interestingly, we found that the block is not at the steps of receptor binding or low pH that triggers conformational changes of viral fusion proteins required for membrane fusion. Rather, we discovered that the creation of hemifusion, an intermediate in which the outer membranes of the two lipid bilayers have merged but the inner membranes still remain intact is blocked by IFITM proteins. We further demonstrated that overexpression of IFITM proteins rigidify the cell membrane, thereby reducing membrane fluidity and fusion potential. Our study provides novel insight into the understanding of how IFITM proteins restrict viral entry and infection.

## Introduction

The interferon (IFN) system is the first line of host defenses against pathogen invasion, including viral infections. It protects by producing hundreds of IFN-stimulated genes (ISGs) that modulate diverse biological functions. A number of ISGs (such as PKR, RNase L, ISG 15, etc.) have been characterized and shown to suppress viral replication, the mechanisms of which are still poorly defined (reviewed in reference [1]). One exciting development in the last few years has been the discovery of some novel ISGs, also known as cellular restriction factors (such as APOBEC3G, Trim5α and Tetherin, etc.), which intrinsically block different steps of retroviral replication [2], [3], [4], [5], [6]. It is notable that many viruses, including retroviruses, have evolved to acquire a variety of strategies that evade IFN-mediated restrictions [7], [8], [9]. This type of intrinsic immunity is believed to play crucial roles in virus-host co-evolution and viral pathogenesis [7], [10].

The interferon-inducible transmembrane (IFITM) protein family belongs to a group of small ISGs (∼15 kD) that has recently been shown to block early stages of viral replication [11], [12]. Originally identified through RNAi genetic screening and shown to inhibit infections by influenza A virus (IAV), West Nile virus and Dengue virus, the IFITM proteins are now known to potently restrict entry and infections by a number of highly pathogenic viruses, including HIV-1, filovirus, and SARS coronavirus [12], [13], [14], [15], [16], [17], [18]. In humans, there are at least 4 functional members of IFITM proteins; IFITM1, 2 and 3 are expressed in a variety of human tissues and cell lines, IFITM5 is limited to the bone and is involved in mineralization [11]. All of these human IFITM proteins have been shown to restrict viral entry and infection, with IFITM3 being generally thought to be the most potent [12], [13], [14], [15], [17], [18]. A recent study demonstrated that the IFITM3 protein significantly restricts the morbidity and mortality associated with influenza, further underscoring the crucial role of IFITM3 *in vivo* [19]. Yount and colleagues recently showed that the mouse IFITM3 protein is not only palmitoylated but also ubiquitinated, and that these posttranslational modifications distinctly regulate the cellular localization of IFITM3 and its anti-influenza activities [20], [21]. While it has been suggested that viral membrane fusion may be blocked by IFITMs [12], [13], [15], direct evidence is still lacking and exactly how IFITM proteins restrict virus entry and infection is currently not known.

Membrane fusion is an essential step for enveloped viruses to enter host cells and initiate infection, a process that is mediated by the viral fusion proteins present on the surface of virions [22]. To prevent premature activation, viral fusion proteins in the mature viral particles are normally metastable and exist at a high-energy state. Once triggered by specific cellular stimuli, such as receptor binding, a low pH, or both, they undergo a series of conformational changes, resulting in the insertion of the fusion peptide of the viral fusion protein into the target cell membrane, leading to hemifusion, pore formation, expansion, and ultimately, complete fusion [23], [24], [25], [26]. While the general principle of viral membrane fusion has been extensively studied, the detailed molecular mechanisms governing this process are still poorly defined [22]. In particular, how viral membrane fusion is modulated by cellular factors other than the specific triggers (such as receptor binding, low pH, cathepsin cleavage, etc.) remains an emerging subject that needs to be explored.

In this work, we sought to determine the mechanisms by which cellular IFITM proteins restrict viral membrane fusion and entry. We chose the Jaagsiekte sheep retrovirus (JSRV) envelope (Env) and IAV hemagglutinin (HA) proteins as the model system of study because of some of their advantages. JSRV is a simple retrovirus, with Env-mediated membrane fusion and entry requiring both receptor-binding and low pH; an initial receptor binding primes the subsequent low pH-dependent conformational changes required for full activation [27], [28], [29], [30]. This unconventional two-step triggering mechanism was originally discovered in the avian sarcoma leukosis virus (ASLV) [31], and has now been suggested to operate in other enveloped viruses, including HCV [32]. Compared to most pH-dependent viruses, JSRV has a relatively high pH threshold (∼pH 6.3) for fusion, the process of which likely occurs in a GPI-anchored-protein-enriched endosomal compartment (GEEC) or caveolae [27], [33], [34]. Thus, study of JSRV Env-mediated fusion should lead to new insights into the mechanism of action of the IFITM proteins. IAV is a prototype pH-dependent virus, the entry and infection of which has been shown to be significantly restricted by IFITM proteins, particularly IFITM3, both *in vitro* and *in vivo* [12], [13], [19]. In addition to JSRV Env and IAV HA, which belong to class I fusion proteins, we also explored the inhibitory effects of IFITM proteins on membrane fusion induced by the Semliki Forest virus (SFV) E1/E2 and vesicular stomatitis virus (VSV) G proteins, which represent class II and III viral fusion proteins, respectively [22]. Hence, the mechanisms uncovered from this study are likely applicable to other viral fusion proteins, and collectively provide critical new insight into our understanding the mechanism by which IFITM proteins restrict viral membrane fusion and entry.

## Results

### Overexpression of IFITM proteins differentially restricts JSRV, IAV, 10A1 MLV and VSV entry

**Figure 1:**
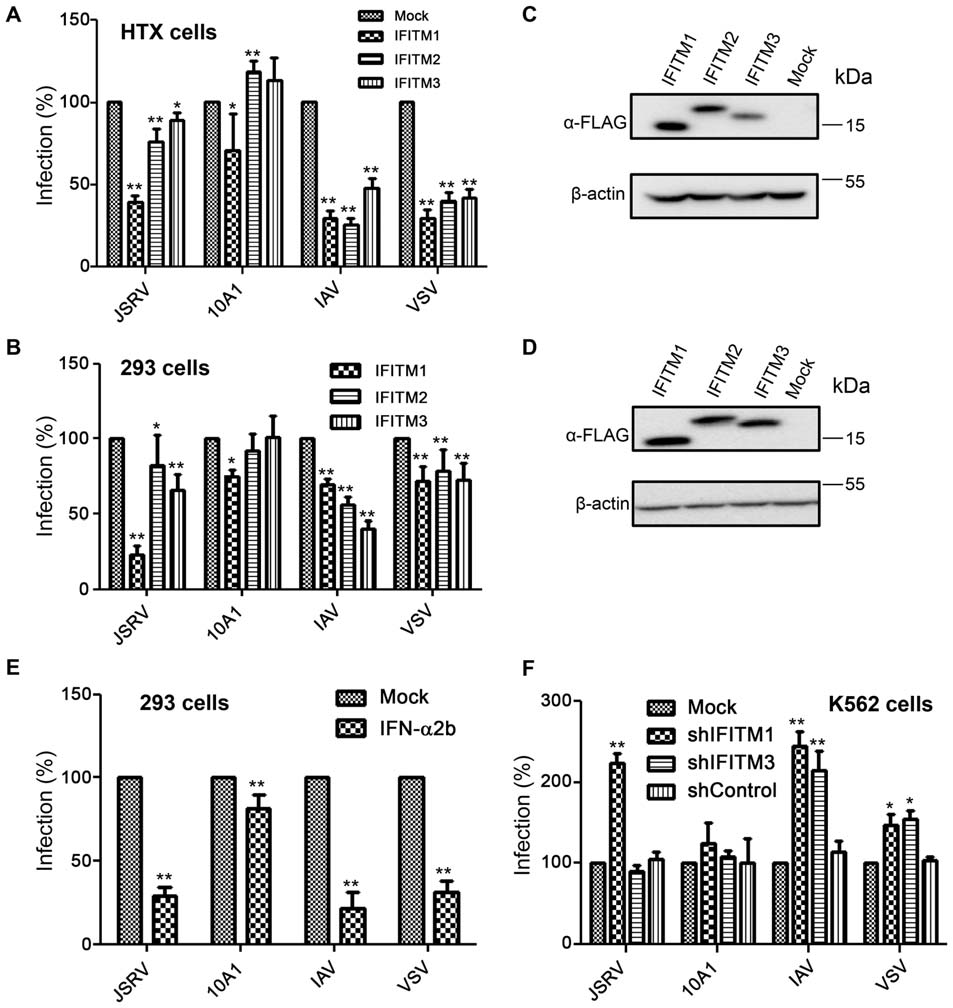
IFITM proteins differentially restrict JSRV, 10A1 MLV, IAV and VSV entry. (**A**) HTX cells stably expressing IFITM1, 2 or 3 were infected with indicated MLV pseudovirions encoding alkaline phosphatase (AP). Three days after infection, the infected cells were fixed and stained for AP activity. Foci of AP-positive cells were counted and normalized to those of parental HTX cells infected with same amounts of pseudovirions (Mock). (**B**) 293 cells stably expressing IFITM1, 2 or 3 were infected with MLV-GFP pseudovirions bearing indicated viral glycoproteins. Two days after infection, the pseudovirus infectivity was determined by flow cytometry; the percents of infection were normalized to those of mock controls. (**C, D**) Immunoblotting of cell lysates harvested from the HTX (C) and 293 (D) cells employed in experiments shown in (A) and (B). Anti-FLAG and anti-β-actin were used as primary antibodies to detect IFITMs and β-actin, respectively. (**E**) 293 cells were treated with 500 units of IFN-α2b for 24 h, and infected with indicated MLV-GFP pseudovirions. The viral infectivity was normalized to that of cells in absence of the IFN-α2b treatment. (**F**) K562 cells stably expressing control shRNA or shRNA targeting IFITM1 or 3 [13] were infected with MLV-GFP pseudovirions bearing indicated viral glycoproteins. The infectivity was measured by flow cytometry and normalized to that of parental K562 cells (Mock) infected with same amounts of indicated pseudovirions. Typically, an MOI of 0.05–0.2 were used for all GFP pseudovirion infections. In all cases, averages ± SD of at least three independent experiments are shown; * denotes p<0.05; ** denotes p<0.01.

Prior studies focused on IFITM3, and have suggested that it mainly acts in late endosomes or lysosomes to restrict viruses that fuse at lower pH (∼pH 5.5) than is present in early endosomes [12], [13], [14], [18]. Here we examined if IFITM proteins also restrict entry of JSRV, whose Env-mediated membrane fusion readily occurs at pH 6.3 or even higher [27], [33]. We did so by creating several stable lines expressing human IFITM1, 2 or 3 and testing their effects on JSRV entry, along with that of several other viruses. We observed that all three IFITM proteins effectively inhibited the infections of MoMLV pseudovirions bearing either IAV HA/NA or VSV-G in HTX cells (a subclone of the HT1080 cell line), with approximately equivalent efficiency (Fig. 1A; p<0.01). Interestingly, the JSRV pseudovirion infection in HTX cells was inhibited by IFITM1 (p<0.01) to a much greater extent than by IFITM2 and 3 (p<0.01 and p<0.05, respectively) (Fig. 1A). IFITM1 also moderately, but consistently, inhibited the infection by amphotropic 10A1 MLV pseudovirions (p<0.05), yet IFITM2 and 3 did not inhibit and even somewhat enhanced entry (Fig. 1A). Similar results were also obtained in 293 cells, where IFITM1 caused the greatest restriction of JSRV entry (p<0.01), although the overall restriction efficiency of these IFITM proteins on VSV and IAV entry was relatively low (Fig. 1B), consistent with a previous report [13]. Immunoblotting revealed that all three IFITM proteins were expressed in both HTX and 293 cells (Figs. 1C and D), with IFITM3 exhibiting a relatively low level of expression in HTX cells (Fig. 1C), which might have contributed to its relatively low antiviral activities in this cell line.

To ascertain that the observed phenotypes of IFITM proteins on viral entry was not due to the FLAG sequences attached to their N-termini, we created HTX cells stably expressing wildtype (WT) IFITM proteins. We observed similar patterns of restrictions by IFITM1, 2 and 3 on all viral pseudotypes tested (Fig. S1). Altogether, these results demonstrate that these three human IFITM proteins effectively restrict IAV and VSV entry, with similar efficiency, while IFITM1 predominantly restricts JSRV entry as compared to that of IFITM2 and 3.

### Stimulation of cells with IFN or depletion of IFITM expression by shRNA distinctly modulates JSRV, IAV, 10A1 MLV and VSV entry

IFITM proteins are normally expressed in cells at a basal level, yet can be significantly induced by type I and type II IFN [35]. To examine if IFN blocks viral entry, we treated 293 or HTX cells with IFN-α2b, a subclass of type I IFN, and examined its effect on pseudoviral infections. We observed that IFN-α2b significantly inhibited infection of 293 cells by JSRV, VSV and IAV pseudovirions (Fig. 1E; p<0.01; data not shown for HTX cells). Interestingly, entry of 10A1 MLV was also slightly but consistently blocked by the IFN-α2b treatment (Fig. 1E; p<0.05), similar to previous reports [13], [36]. The greater inhibitory effect of IFN on IAV and VSV entry into 293 cells (Fig. 1E) relative to that of overexpressing individual IFITM proteins (Fig. 1B) was unexpected because IFITM proteins had higher levels when overexpressed than when induced by IFN (Fig. S2). Perhaps IFITMs induced by IFN synergistically cooperated to inhibit viral entry; additionally, ISGs other than IFITMs might have contributed to the observed effects of IFN treatment in 293 cells. No cytotoxicity was observed for the doses of IFN applied during the viral infection period.

Given that HTX or 293 cells do not express a significant level of endogenous IFITMs, especially IFITM1 and 3 (Fig. S2), we next used a myelogenous leukemia line, K562 cells, to address if depletion of IFITM expression would enhance viral entry. We observed that, indeed, the entry of JSRV and IAV, and to a lesser extent that of VSV, was enhanced in K562 cells stably expressing shRNA against IFITM1 or 3 (kind gifts of Michael Farzan and I-Chueh Huang, Harvard Medical School) [13] as compared to the parental K562 cells (Fig. 1F; p<0.01 or 0.05). The entry of 10A1 MLV was also slightly enhanced by shRNA targeting IFITM1, but the increase was not statistically significant (Fig. 1F; p>0.05). shRNA did not significantly reduce IFITM2 in K562 cells, and thus we could not assess the consequences of reducing of this protein (data not shown). Overall, these results suggest that endogenous IFITM proteins intrinsically restrict JSRV, IAV and VSV entry.

### IFITM expression does not affect binding of JSRV Env to its Hyal2 receptor or receptor-mediated priming for fusion activation

**Figure 2:**
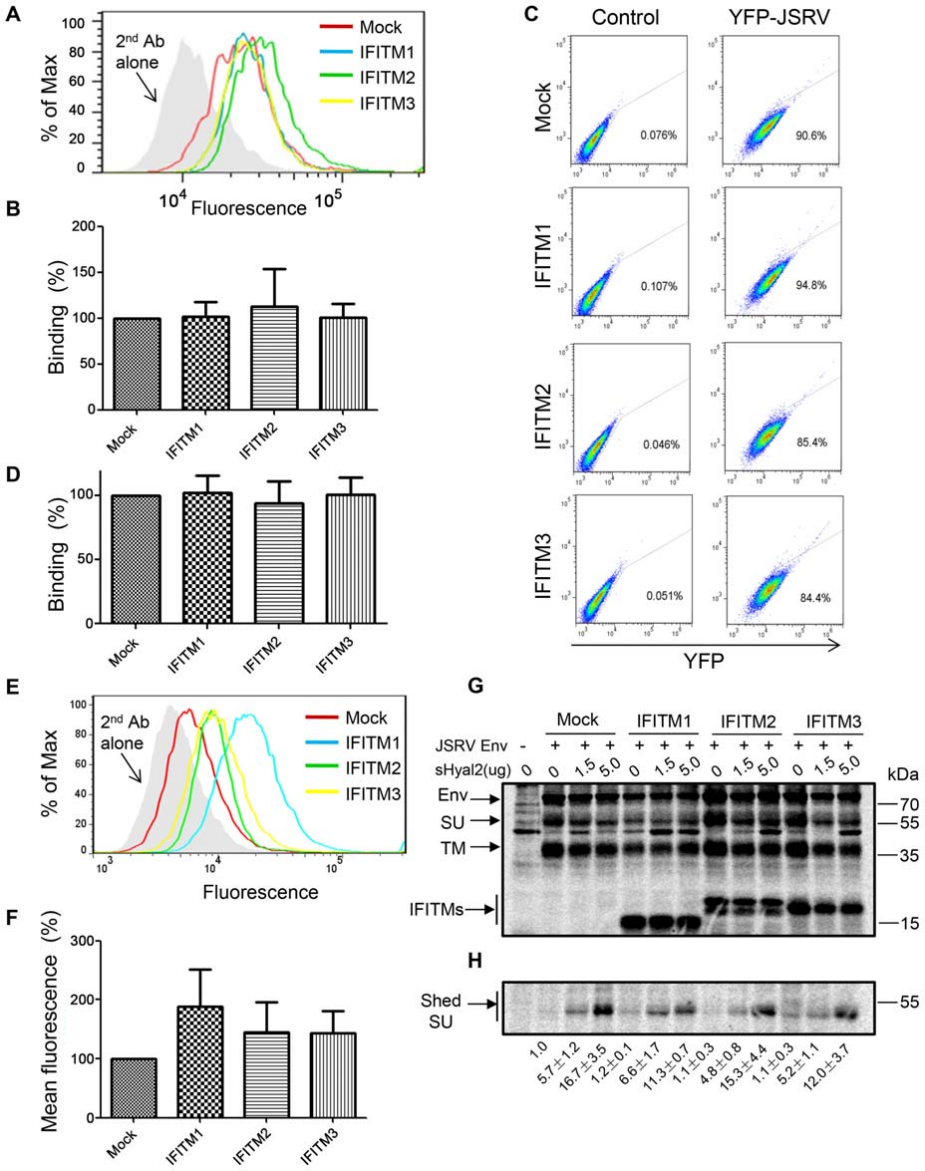
Expression of IFITM proteins does not affect binding of JSRV Env to cells expressing the Hyal2 receptor, nor does it perturb receptor-mediated priming for fusion activation. (**A**) Examination of JSRV Env binding to cells expressing IFITMs. HTX cells stably expressing indicated IFITM proteins were incubated with purified JSRV SU-human IgG Fc proteins at 4°C; following incubation with FITC conjugated anti-human Fc antibody, cells were analyzed by flow cytometry. Representative histograms from one typical experiment are shown. Arrow indicates the secondary antibody alone control. (**B**) Quantitative analysis of JSRV Env binding data shown in (A). The fluorescence intensities (geometric means) obtained from (A) were averaged and normalized to those of mock controls. The means ± SD of at least three independent experiments are shown. (**C**) Examination of the JSRV pseudovirion binding to cells expressing IFITMs. HTX cells expressing IFITM proteins were incubated with purified JSRV pseudovirions containing MLV Gag-YFP to allow virus binding. Cells were washed, fixed and analyzed by flow cytometry. “Control” indicates cells incubated with MLV Gag-YFP pseudovirions in the absence of JSRV Env. Representative flow cytometry profiles are shown. (**D**) Quantitative analysis of the JSRV pseudovirion binding experiments shown in (C). The fluorescence intensities of three independent experiments were averaged and plotted. (**E**) Expression of IFITM proteins on the surface of HTX cells. The expression was examined by an anti-FLAG antibody and analyzed by flow cytometry. (**F**) Quantitative analysis of IFITM expression on the cell surface shown in (E). Values are the means ± SD of at least five independent experiments. (**G and H**) Examination of the effect of IFITMs on JSRV SU shedding. 293T cells were co-transfected with plasmids encoding FLAG tagged-JSRV Env and FLAG-tagged IFITMs. Cells were metabolically labeled and chased in the presence of indicated amounts of sHyal2. Cell lysates and culture media were harvested and immunoprecipitated with anti-FLAG beads. Samples were resolved by SDS-PAGE and subjected to autoradiograthy. (G) Expression of JSRV Env and IFITM in transfected cells. Env: the full length of JSRV Env; SU: surface subunit; TM: transmembrane subunit. (H) Shedding of JSRV SU into culture medium. Note the increased SU shedding in cells expressing JSRV Env with increasing amounts of sHyal2; no significant differences in shedding among cells expressing IFITM and mock controls were observed. The relative intensities of signals for shed SU were calculated by setting the signals of the mock control without sHyal2 stimulation as 1.0; three independent experiments were used for the quantification.

IFITM1 has been previously shown to be associated with caveolin-1, a protein that is known to play an essential role in caveolin-mediated endocytosis [37], [38]. Given that the JSRV receptor, hyaluronidase 2 (Hyal2), is a GPI-anchored protein that is localized in lipid rafts and that JSRV may use a GPI-anchored-protein-enriched endosomal compartment (GEEC) and/or a caveolar pathway for entry [33], [34], [39], [40], we considered the possibility that IFITM1 could preferentially interfere with the binding of JSRV Env to Hyal2, thereby restricting viral entry. We took advantage of a soluble form of the JSRV SU-human IgG fusion protein (JSU-hFc) we previously created, and performed an *in vitro* binding assay on HTX cells expressing individual IFITM proteins and functional human Hyal2 [27], [28], [29], [41]. Flow cytometry analysis revealed that the fluorescence shifts in HTX cells expressing IFITM proteins, including that of IFITM1, were similar to those of parental cells (Figs. 2A and B). This indicates that expression of IFITM proteins did not affect the binding of JSRV Env to HTX cells expressing the Hyal2 receptor. We also performed virus binding assays using Gag-YFP-expressing MoMLV (kind gifts of Walter Mothes) pseudoviral particles bearing JSRV Env [28], [42]; again, similar fluorescence intensities were observed among cells expressing IFITM proteins and parental cells (Figs. 2C and D). The expression of IFITM proteins on the surface of HTX cells was also examined by using an anti-FLAG antibody. IFITM1 had a relatively higher level of surface expression as compared to IFITM2 and 3, but overall the fluorescence signals were low and their differences were not statistically different (Figs. 2E and F; data not shown). We conclude that expression of IFITM proteins, including IFITM1, does not affect the binding of JSRV Env to its Hyal2 receptor on the cell surface.

JSRV Env uses a dual triggering mechanism in which receptor binding primes the Env to undergo low pH-dependent conformational changes that lead to fusion [28], [29]. This unusual feature allowed us to examine if IFITM proteins may affect receptor-mediated priming for fusion. We performed metabolic labeling of 293T cells co-expressing IFITMs and JSRV Env, and determined shedding of JSRV SU in the presence or absence of a soluble form of Hyal2 (sHyal2). In our previous studies, we had established that shedding of JSRV SU into culture media is an important indicator of Hyal2 receptor-mediated triggering for the fusion activation of JSRV Env [28], [29], [30]. Here we observed that the levels of JSRV SU harvested from the culture media of 293T cells expressing IFITM1, 2 or 3 were comparable to those of parental cells, and that they all increased with the presence of sHyal2 in a dose-dependent manner (Fig. 2H). The total levels of JSRV Env expression in these radiolabeled cells were approximately equivalent, as evidenced by the intensities of Env precursors and processed TMs (Fig. 2G). Collectively, we conclude that overexpression of IFITM proteins, including IFITM1, does not affect the expression and trafficking of JSRV Env, nor does it impair receptor-mediated priming for fusion activation.

### IFITM1 profoundly inhibits syncytia formation induced by JSRV Env and IAV HA; the effect occurs over a broad range of pH

**Figure 3:**
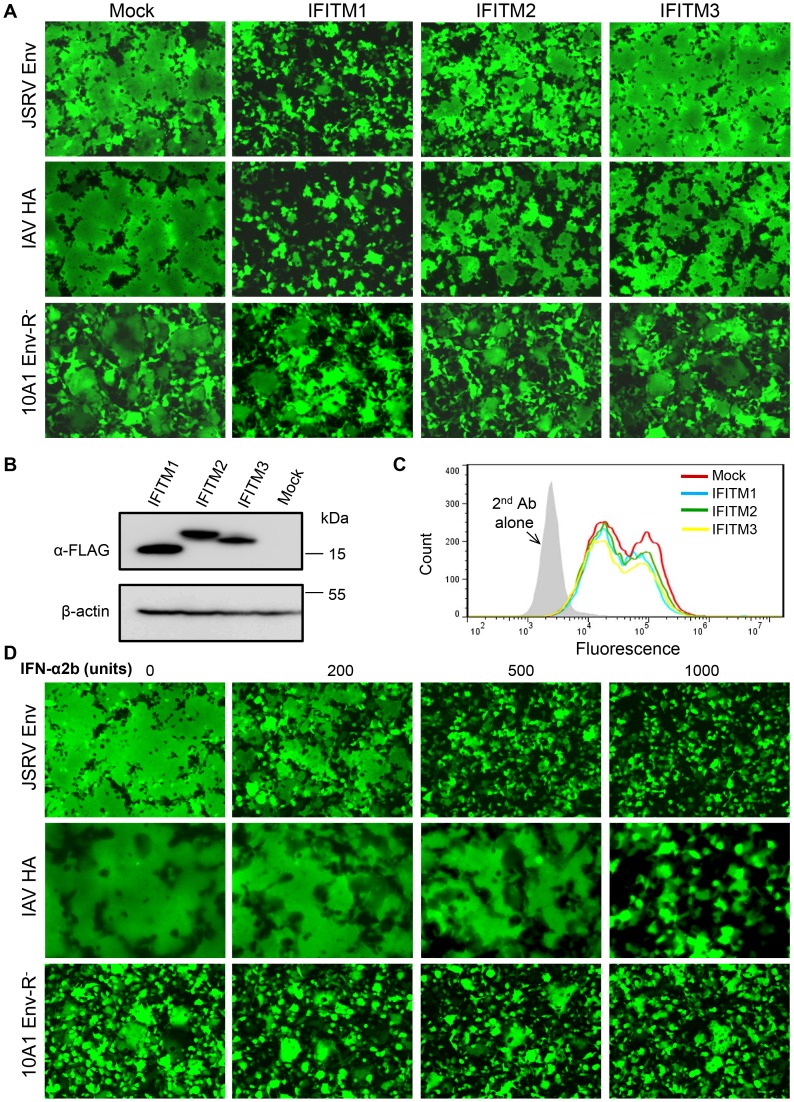
Expression of IFITM proteins or treatment of cells with IFN suppresses syncytia formation induced by JSRV Env and IAV HA. (**A**) 293/LH2SN cells stably expressing the indicated IFITM proteins were transfected with plasmid DNA encoding JSRV Env, IAV HA or 10A1 MLV Env with the R peptide deleted (10A1 Env R^−^); cells were treated with a pH 5.0 buffer for 1 min (for JSRV Env and IAV HA) or left untreated (for 10A1 MLV Env) and analyzed for syncytium formation using fluorescence microscopy. Note the stronger inhibition of IFITM1 relative to that of IFITM2 and 3 for JSRV Env and IAV HA; no apparent inhibition was observed for 10A1 MLV Env. (**B**) Expression of IFITM proteins in 293/LH2SN cells was determined by immunoblotting with an anti-FLAG antibody. β-actin was used as a loading control. (**C**) Expression of IFITM proteins does not downregulate the JSRV Env expression on the cell surface. 293T cells were co-transfected with plasmid DNAs encoding FLAG-tagged JSRV Env (at both the N- and C-termini) plus indicated wildtype IFITMs. Cells were incubated with an anti-FLAG antibody on ice, and the surface expression of JSRV SU was determined by flow cytometry. A second antibody alone was used control. (**D**) 293/LH2SN cells were transfected with plasmids encoding JSRV Env, IAV HA, or 10A1 MLV Env with the R peptide deleted; 6 h after transfection, cells were treated with indicated amounts of IFN-α2b or medium for 24 h. Cells were exposed to a pH 5.0 buffer for 1 min (for JSRV Env and IAV HA) or left untreated and examined for syncytia formation.

Syncytia formation and cell-cell fusion assays have been instrumental in understanding membrane fusion, including viral membrane fusion [43]. We sought to obtain direct evidence that IFITM proteins may restrict viral membrane fusion mediated by JSRV Env and other viral fusion proteins. As JSRV Env requires Hyal2 overexpression for membrane fusion to be detected at low pH [27], we generated stable HTX and 293 cell lines overexpressing Hyal2 and IFITM1, 2 or 3, which served as target cells for the syncytia formation and cell-cell fusion assays described below. For parental 293 cells overexpressing Hyal2 (293/LH2SN, mock), we observed almost complete syncytia formation (∼100%) induced by JSRV Env and IAV HA within 5–10 min after a pH 5.0 pulse (Fig. 3A). In sharp contrast, very little syncytia formation was detected in 293/LH2SN cells expressing IFITM1, even after a 1 h recovery period (Fig. 3A). Syncytia formation was also substantially reduced in 293/LH2SN cells expressing IFITM2, but the reduction was much less in cells expressing IFITM3, especially in the case of JSRV Env (Fig. 3A). There is much less or no inhibitory effect of these IFITM proteins on entry of 10A1 MLV (a virus that fuses at neutral pH, Fig. 2B) [12], [13]. We therefore measured syncytia formation induced by 10A1 MLV Env (with its R peptide deleted) at neutral pH and found, as predicted, that it was not significantly affected by IFITMs (Fig. 3A). The fusion efficiency of 10A1 MLV Env in IFITM1, 2 and 3-expressing cells, as quantified using fusion index (0.51±0.06, 0.50±0.05 and 0.52±0.04, respectively), was comparable to that in parental cells (0.52±0.06). A similar order of syncytia inhibition by the IFITMs, i.e., IFITM1>IFITM2>IFITM3, on JSRV Env and IAV HA was also obtained in cells expressing WT IFITM proteins (without the N-terminal FLAG tags) (Fig. S3). The differential inhibitory effects of IFITMs on syncytia formation of JSRV Env and IAV HA in 293/LH2SN cells were unlikely due to their levels of IFITM expression, which were examined by Western blots (Fig. 3B). Flow cytometry analysis of 293T cells co-expressing JSRV Env and WT IFITM proteins showed that the levels of JSRV Env on the surface of IFITM-expressing cells were comparable to that of the mock control (Fig. 3C), indicating that the reduced syncytia formation was not due to a change in the Env surface expression.

To evaluate if the differential effects of IFITM proteins on syncytia formation induced by JSRV Env were dependent over a limited pH range, we treated the JSRV Env-expressing cells with different pH values, i.e., pH 6.2, 5.7 and 5.0, respectively. Under these pH conditions, IFITM1 consistently exhibited the strongest inhibition on syncytia formation induced by JSRV Env (Fig. S4). Further lowering the pH (pH 4.0) or incubating 293 cells with an increased concentration of sHyal2 (up to 30 µg/ml) did not overcome the IFITM1-mediated restriction on fusion (data not shown), suggesting that the block by IFITM1 does not occur at the triggering step. It has been previously established for influenza HA that progressively lowering pH causes the activation of more fusion proteins; rather than causing each individual protein to undergo increasingly extensive conformational changes [44], [45]. Based on the results of JSRV Env described here, we conclude that the mechanism of IFITM inhibition is independent of fusion protein density.

We also assessed if treatment of cells with IFN could suppress viral membrane fusion. We observed that, following a 24-h incubation of 293/LH2SN cells with IFN-α2b, syncytia formation induced by JSRV Env or IAV HA was greatly reduced in a dose-dependent manner (Fig. 3D). In contrast, 10A1 MLV Env-mediated syncytia formation was not significantly affected by the IFN-α2b treatment (Fig. 3D). No cytotoxicity was observed during the 24-h IFN treatment period; nor were there any changes in the expression of JSRV Env and Hyal2, as examined by immunoblotting and flow cytometry (data not shown). Since 293 cells do not express significant amounts of IFITMs, especially IFITM1 and 3 (Fig. S2), we have been unable to unambiguously determine if depletion of individual endogenous IFITMs by shRNA in 293 cells enhances syncytia formation. Nonetheless, these experiments clearly demonstrated that IFN can block viral membrane fusion.

### Cell-cell fusion induced by JSRV Env is inhibited by IFITM1; inhibition is the same for expression in target as in effector cells

**Figure 4:**
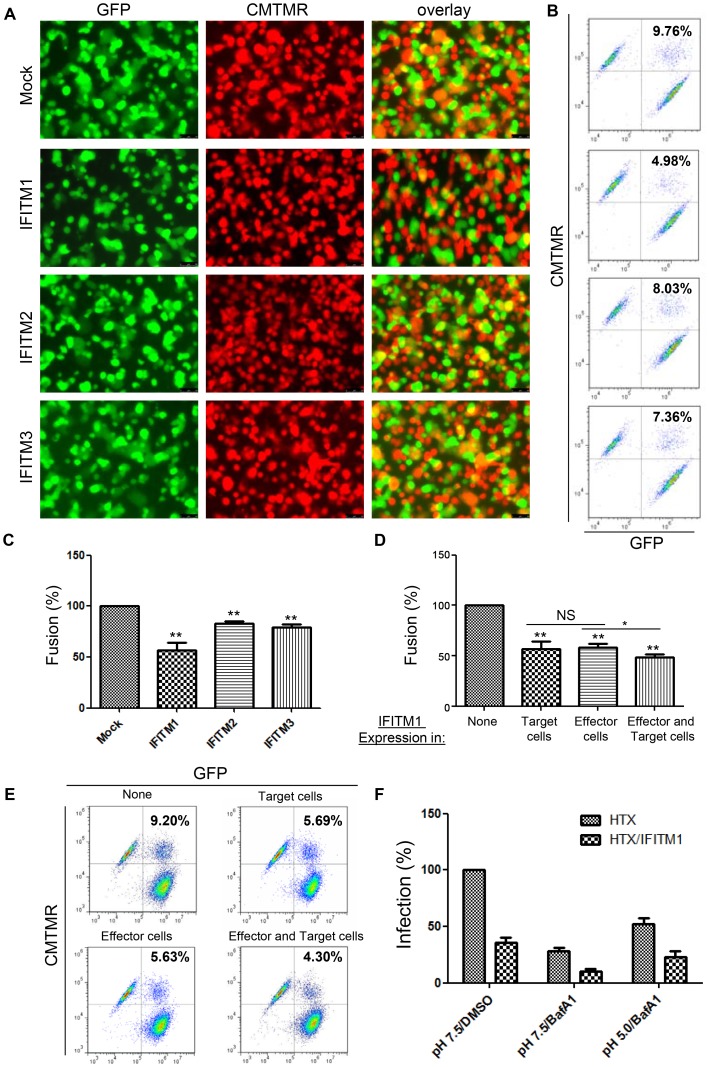
Cell-cell fusion induced by JSRV Env is inhibited by IFITM proteins, and extracellular low pH pulse cannot overcome IFITM1-mediated restriction on JSRV entry. Effector 293T/GFP (green) cells were transfected with a plasmid encoding JSRV Env. The next day, cells were co-cultured with CMTMR (red)-labeled HTX/LH2SN cells stably expressing indicated IFITM proteins. Co-cultured cells were then treated with a pH 5.0 buffer for 1 min, and cell-cell fusion was examined by fluorescence microscopy (**A**) and flow cytometry (**B**). Values shown in the upper-right quadrant of flow cytometry profiles represent percentages of fused cells. (**C**) Percentages of fused cells in HTX/LH2SN cells expressing IFITMs were normalized to those of mock controls. Values are the means ± SD of at least three independent experiments. (**D and E**) Effector 293T/GFP cells were transfected with a JSRV Env-encoding plasmid alone (columns 1 and 2) or plasmids encoding JSRV Env plus IFITM1 (columns 3 and 4); 24 h post-transfection, cells were co-cultured with CMTMR-labeled target cells, either parental HTX/LH2SN (columns 1 and 3, Mock) or HTX/LH2SN stably expressing IFITM1 (columns 2 and 4). Percents of fused cells were measured by flow cytometry (E), averaged, and normalized to those of controls (column 1) (D). Values are the means ± SD of at least three independent experiments. * p<0.05; ** indicates p<0.01. NS: not statistically significant (p>0.05). (**F**) Low pH does not overcome IFITM1-mediated block of JSRV entry. HTX or HTX cells expressing IFITM1 were pretreated with 20 nM BafA1 (middle and right columns) or with 0.01% DMSO (left columns, as controls) for 2 h and then spininoculated with JSRV pseudovirions at 4°C for 1 h. Cells were washed with cold PBS to remove unbound viruses before incubation at 37°C for 1 h to allow endocytosis (see references 33 and 34). Cells were then treated with either a pH 7.5 or pH 5.0 buffer at 37°C for 5–10 min, followed by an additional incubation with 0.01% DMSO or 20 nM BafA1 for 4 h to allow infection. Three days after infection, viral titers were determined by counting AP-positive foci, and relative infectivity was calculated by normalizing all titers relative to those in parental HTX cells treated with DMSO. Values are the means ± standard deviations of three independent experiments.

We applied a more quantitative cell-cell fusion assay to evaluate the effects of IFITM proteins on JSRV Env-mediated fusion as well as to understand the possible mechanisms for inhibition. We expressed JSRV Env in 293T effector cells stably expressing GFP, and labeled HTX/LH2SN target cells expressing IFITM proteins with a red-fluorescent dye, CMTMR; cell-cell fusion was measured by a fluorescence microscope and flow cytometry [27]. Similar to the syncytia formation results (Fig. 3A), IFITM1 exhibited the strongest inhibition, reducing the cell-cell fusion efficiency of JSRV Env by ∼50% (Figs. 4A, B and C; p<0.01). IFITM2 and 3 also suppressed cell-cell fusion, but with much less efficiency (Figs. 4A, B and C; p<0.01). Given that the sizes of fused cells in parental HTX/LH2SN cells were much larger than those of fused cells in IFITM1-expressing cells (Fig. 4A), and that the cell populations with larger size were excluded from flow cytometry analysis, it is likely that the percent of fusion reduction measured by flow cytometry (Figs. 4B and C) underestimates the inhibitory effect of IFITM proteins (see another cell-cell fusion assay below). Nevertheless, these experiments provide clear indication that the content transfer resulting from cell-cell fusion by JSRV Env was inhibited by IFITM proteins.

We explored whether IFITM proteins expressed in effector cells co-expressing the viral fusion proteins could also restrict cell-cell fusion. We expressed IFITM1 in 293T/GFP effector cells, in HTX/LH2SN target cells (the stable cell lines described above), or in both, and compared their effects on JSRV Env-induced cell-cell fusion. Expressing IFITM1 in effector cells (Fig. 4D, column 3; Fig. 4E) was as effective in reducing cell-cell fusion by JSRV Env as expressing IFITM1 in target cells (Fig. 4D, column 2; Fig. 4E), and co-expressing IFITM1 in both effector and target cells enhanced this inhibitory effect (Fig. 4D, column 4; compare columns 2 and 3 with column 4; p<0.05 in both cases; Fig. 4E). Thus, the inhibition of cell-cell fusion by IFITM1 is not specifically related to its expression in effector or target cells. This result suggests that IFITM proteins are unlikely to suppress cell-cell fusion by directly acting on specific viral fusion proteins or their corresponding receptors, but rather through a common physical mechanism(s) (see below).

A critical question is whether or not the cell-cell fusion assay employed here is relevant to endosomal fusion; this is particularly important, given that IFITM proteins have been shown to predominantly restrict viruses that require low pH for membrane fusion and entry. To address this question, we took advantage of our previous finding that JSRV pseudovirions are virtually resistant to low pH inactivation and that an extracellular low pH pulse can overcome proton pump inhibitor–mediated block of JSRV entry [27], [33], [34]. We pretreated JSRV pseudovirion-bound HTX or HTX/IFITM1 cells with 20 nM bafilomycin A1 (BafA1), followed by a pH-5.0 pulse for 5–10 min; cells were then allowed for infection for 4 h in the presence of BafA1. We observed that, while low pH substantially rescued the BafA1-mediated block in both the parental HTX and HTX/IFITM1 cells, as would be expected [33], [34], the low pH treatment did not increase the JSRV titer in HTX/IFITM1 cells to a level that was similar to that of parental HTX cells (Fig. 4F). This result apparently differed from that of SARS coronavirus, whose fusion inhibition by IFITMs had been previously shown to be bypassed by trypsin [13]. Overall, our data suggest that IFITM1 expressed on the plasma membrane effectively blocks the forced entry of JSRV rendered by the low pH pulse, and this is consistent with the cell-cell fusion data.

### IFITM proteins suppress cell-cell fusion induced by all three classes of viral fusion proteins; the inhibition efficiency can be cell type dependent

**Figure 5:**
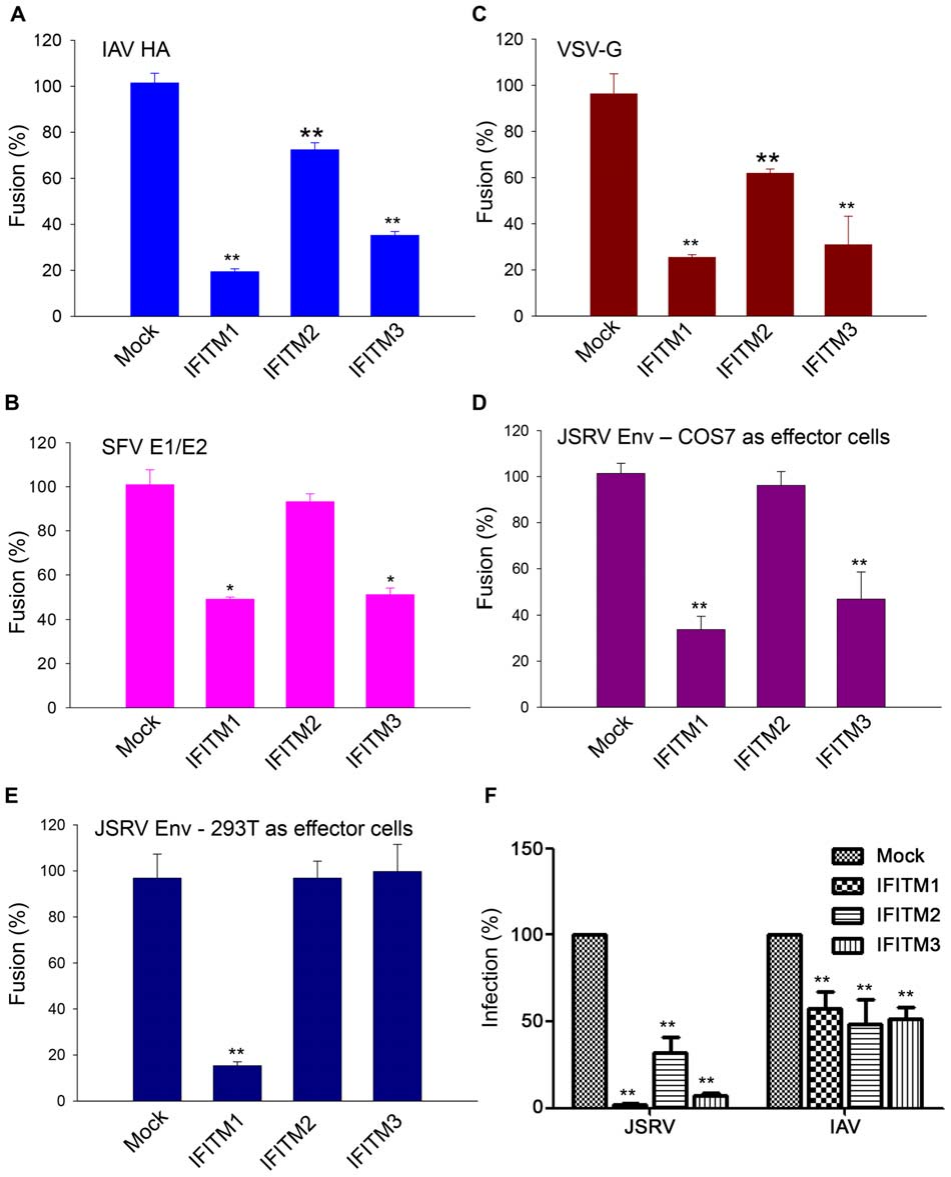
IFITM proteins inhibit cell-cell fusion induced by representatives of three classes of viral fusion proteins. (**A to E**) Effector cells expressing indicated viral fusion proteins were loaded with calcein-AM, and were bound to target 293/LH2SN cells (Mock) or to cells expressing indicated IFITM proteins that were prelabeled by CMAC. pH was then lowered to 5.0 for JSRV Env, 4.8 for IAV HA, 5.7 for VSV G, and 5.4 for SFV E1/E2. Following reneutralization of cells to 7.2, fusion between pairs of effector and target cells was scored under fluorescence microscopy. (**A**) IAV HA (a class I fusion protein). (**B**) VSV G (class III). (**C**) SFV E1/E2 (class II). (**D**) JSRV Env (class I), with COS7 as effector cells. (**E**) JSRV Env, with 293T cells as effector cells. Note distinct effects of IFITM3 on JSRV fusion shown in (D) and (E). (**F**) Restriction of JSRV and IAV entry by IFITM proteins in COS7/LH2SN cells. COS7/LH2SN cells (Mock) or derivatives expressing indicated IFITM proteins were infected with GFP-encoding MoMLV pseudovirions bearing JSRV Env or IAV HA/NA, and viral infectivity was determined by flow cytometry as described in Fig. 1. Note that IFITM3 inhibited JSRV entry as effectively as did the IFITM1; representative flow cytometry profiles are shown in Fig. S4A.

In order to assess possible broad effects of IFITMs on viral membrane fusion, we employed another cell-cell fusion assay for examination of IAV HA, SFV E1/E2, and VSV-G, which represent class I, II and III fusion proteins, respectively [22]. In these experiments, effector cells were the NIH 3T3-derived HAB2 cell line stably expressing IAV HA (kind gift of Judy White) [46] or COS7 transiently transfected with plasmids encoding SFV E1/E2 or VSV-G; we labeled these cells with calcein-AM. We loaded the target 293/LH2SN cells expressing individual IFITM proteins (the same cell lines as used for the syncytia formation assay shown in Fig. 3) with CMAC, allowing aqueous dye transfer during fusion to be monitored by a fluorescence microscope. We found that, somewhat surprisingly, both IFITM1 and 3 strongly inhibited membrane fusion induced by all three classes of viral fusion proteins, and their efficiencies were almost the same. IFITM2 also inhibited viral membrane fusion, but the efficiency was generally low (Figs. 5A, B and C), consistent with the results of syncytia formation (Fig. 3A) and the GFP-transfer cell-cell fusion assay described above (Fig. 4). Even more surprisingly, we observed that when the JSRV Env protein was expressed in COS7 cells as effector, Env-mediated fusion was also markedly suppressed by IFITM3 (Fig. 5D). This was in sharp contrast to the situation in which IFITM3 had essentially no effect on fusion when 293T cells were used as effector cells to express JSRV Env (Fig. 5E).

Perhaps COS7 cells express a specific cellular factor(s) that functionally promotes the inhibitory effect of human IFITM3 on viral membrane fusion. Consistent with this notion, COS7 cells expressing human IFITM3 (also engineered to co-express human Hyal2, because COS7 cells are not permissive to JSRV infection) drastically suppressed JSRV entry, with efficiency almost equivalent to that of IFITM1 (Figs. 5F and S5A). This observation was in sharp contrast to the situations in HTX and 293 cells, where IFITM3 had much less effect (Figs. 1A and B). Similarly, the syncytia forming activity of JSRV Env, as well as that of IAV HA, was almost equally inhibited by human IFITM3 and IFITM1 in COS7 cells (Fig. S5B) as compared to that in 293/LH2SN cells (Fig. 3A). This cell type-dependent effect on cell-cell fusion mirrored prior reports showing that the IFITM-mediated restriction on viral entry and infection is also cell type dependent [13], [15]. Collectively, our data demonstrated that these three human IFITM proteins potently suppress cell-cell fusion induced by all three classes of viral fusion proteins.

### Chlorpromazine (CPZ) does not overcome the block of cell-cell fusion induced by IFITM proteins

**Figure 6:**
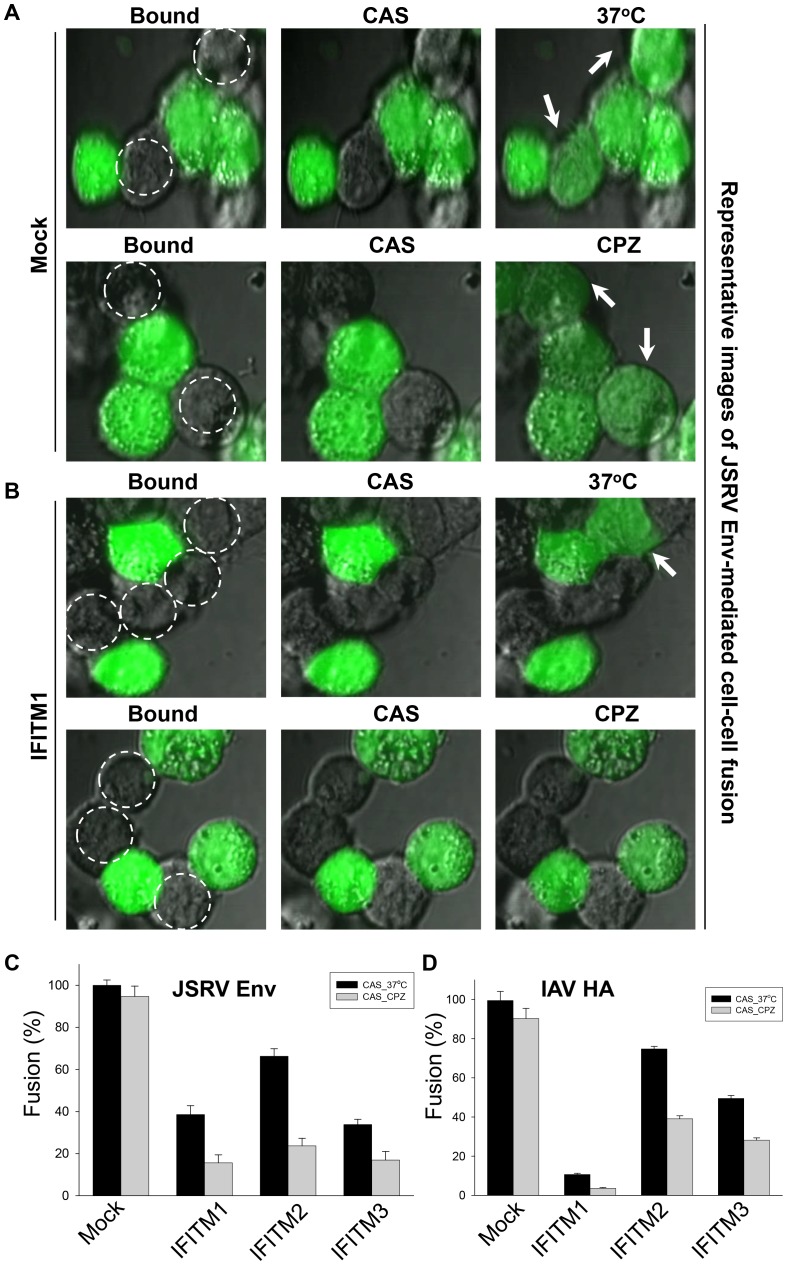
CPZ does not rescue the restriction of IFITMs on cell-cell fusion of viral fusion proteins. COS7 cells expressing JSRV Env or HAB2 cells expressing IAV HA (images not shown) were loaded with calcein-AM (green) and bound to target cells (unlabeled), either parental 293/LH2SN (Mock) or derivatives expressing IFITM1 (IFITM1). Cells were treated with a pH 5.0 buffer at 4°C for 1 min to create a cold arrested state (CAS), at which aqueous dye had not transferred. Cells were then switched to 37°C or treated with CPZ, cell-cell fusion were monitored under a fluorescence microscope. (**A**) In mock cells (JSRV Env-mediated fusion): Upon raising temperature from the 4°C of CAS to 37°C, the two target cells of the image became labeled by calcein-AM (arrows), illustrating that fusion was now extensive. Similarly, adding CPZ to cells at CAS also led two target cells receiving calcein-AM, illustrating that fusion was as extensive upon addition of CPZ as upon raising temperature. (**B**) In IFITM1-expressing cells (JSRV Env-mediated fusion): Raising temperature led to calcein transfer to only one (arrow) of the four target cells. Addition of CPZ did not lead to calcein-AM transfer to any of the three target cells. (**C–D**) The quantifications of these phenomena are presented in JSRV Env (C) and for IAV HA (D). Similar experimental procedures were applied to IFITM2 and 3-expressing cells, and the data were plotted as show in (C) and (D).

We next examined which steps of the viral membrane fusion process are inhibited by IFITM proteins. For this purpose, we used conditions that, in the absence of IFITM proteins, allow fusion to proceed up to and through the point of hemifusion while preventing the steps that lead to pore formation. We did so by creating an intermediate of fusion, referred to as a cold arrested state (CAS). It has been previously shown that for viral fusion proteins that induce fusion at low pH, lowering pH at the low temperature of 4°C yields hemifusion; raising temperature at neutral pH then leads to pore formation and growth [47], [48], [49]. We created CAS for JSRV Env and IAV HA, and found, as expected, that membrane fusion did not occur (Figs. 6A and B, middle panels). Adding CPZ at neutral pH and low temperature to the parental cells (mock) resulted in a significant aqueous dye transfer (Figs. 6A, C, and D). Any further dye transfer upon subsequently raising of temperature to 37°C was not statistically significant (Figs. 6A, C and D). For target cells expressing IFITM proteins, aqueous dye spread upon CPZ addition at CAS was much less than for the parental cells (Figs. 6B, C and D). In contrast, raising the temperature led to a significant increase in dye spread; but the total dye spread was still less than for the parental cells (Figs. 6B, C and D). The fact that the amount of dye spread induced by CPZ was relatively small showed that little hemifusion occurred for the IFITM-expressing cells. The greater amount of dye spread upon raising temperature indicated that the IFITM proteins in the target cell membrane block the creation of hemifusion. Temperature dependence of protein conformational changes is generally greater than for those of lipids that are not near a phase transition. We therefore assume that the presence of IFITM proteins blocked the conformations changes in JSRV Env and IAV HA needed for hemifusion. But it is possible that the decrease in membrane fluidity caused by IFITM proteins (see below) inhibits lipids from rearranging into a hemifusion configurations.

### The negative curvature-promoting lipid, oleic acid (OA), effectively rescues IFITM-mediated suppression of viral membrane fusion

**Figure 7:**
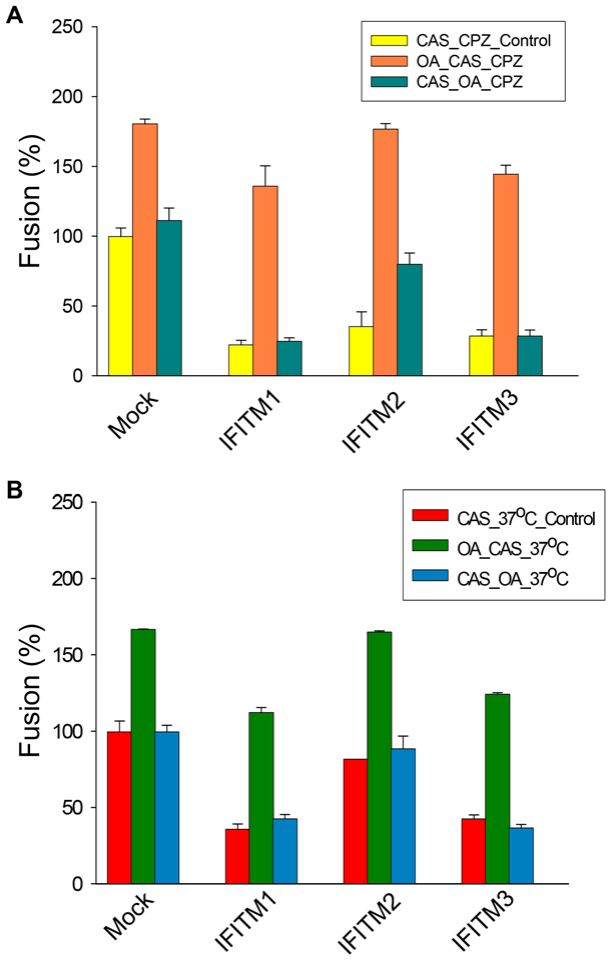
Making spontaneous curvature more negative prior to creating CAS promotes JSRV Env-mediated hemifusion. CAS was created as described in Fig. 6. OA was added prior or subsequent to CAS (see details in Materials and Methods). The addition of OA before creating CAS (middle columns of groups of three) promoted aqueous dye transfer upon either addition of CPZ (**A**) or raising temperature to 37°C from CAS (**B**). In contrast, adding OA after creating CAS (third columns), did not affect the extents of dye transfer caused by either CPZ addition or by raising temperature as compared to control (first columns). This was the general pattern, independent of whether target cells contained indicated IFITM proteins or not (Mock).

Hemifusion is promoted by negative spontaneous curvature of monolayers that contact each other in binding [50], [51]. Therefore, making the spontaneous curvature more negative should oppose the inhibitory actions of IFITMs and thereby promote hemifusion. We tested this by adding oleic acid (OA) to aqueous solutions for 15 min to allow them to incorporate into plasma membranes. We observed that creating CAS in the presence of OA led to more fusion both when adding CPZ (Fig. 7A, compare second columns with first columns in each cell line) and raising temperature (Fig. 7B, compare second columns with first columns in each cell line). For the parental target cells, the addition of OA resulted in fusion between almost all cell pairs (Figs. 7A and B). For target cells expressing IFITM proteins, OA-addition led to a great increase in the percentage of cells pairs that fused (Figs. 7A and B). In sharp contrast, incorporating OA into membranes subsequent to creating CAS was much less effective in promoting fusion upon either the addition of CPZ or the raising of the temperature (Figs. 7A and B, compare third columns with second columns in each cell line). The almost complete rescue of IFITM-mediated restriction on hemifusion by OA further supports the notion that the major block in fusion caused by ITIFM proteins in target membranes occurs upstream of hemifusion. The presence of IFITMs likely blocks hemifusion by making the spontaneous curvature of outer leaflets of plasma membranes more positive.

### Overexpression of IFITM proteins results in increased lipid order of cell membranes

**Figure 8:**
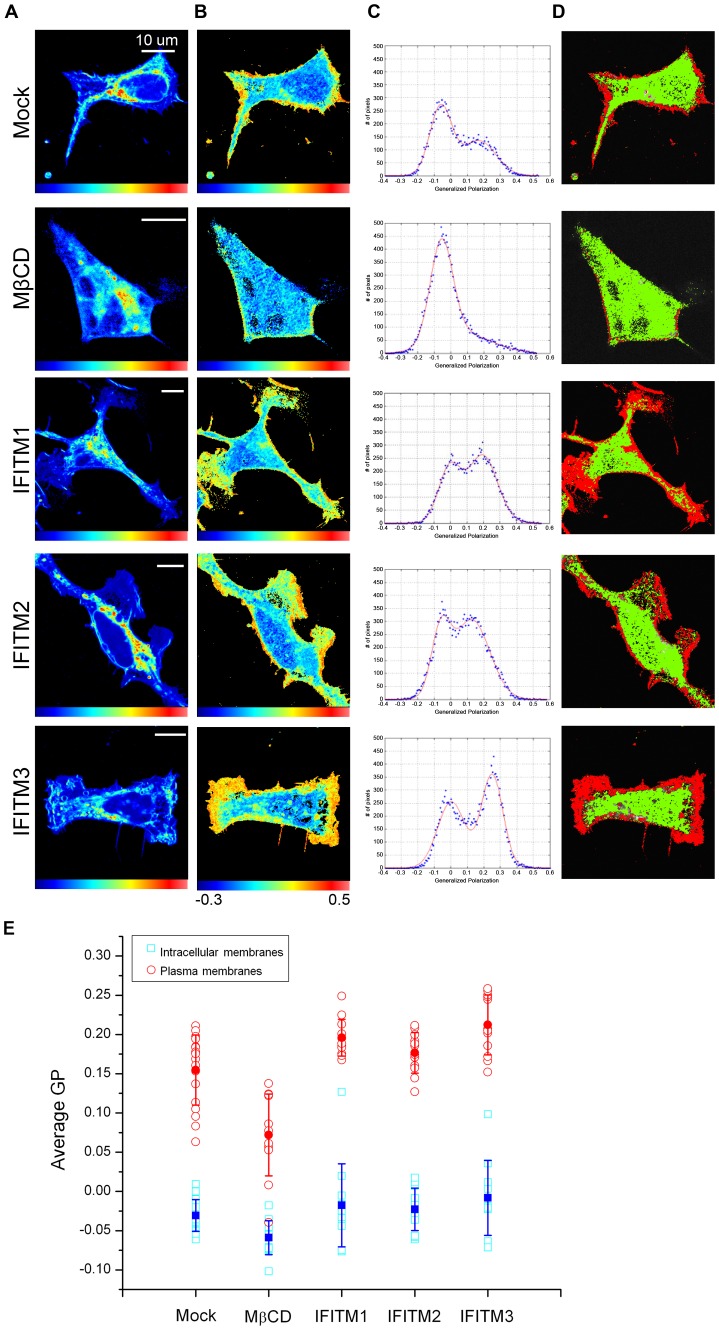
Expression of IFITM proteins increases the lipid order of cell membranes. 293/LH2SN cells stably expressing IFITM1, 2 or 3, or mock controls were incubated with 1.8 µM Laurdan for 40 min at 37°C, and were imaged using two-photon fluorescence microscope. Parental 293/LH2SN cells were treated with 10 mM MβCD for 1 h to serve as controls. (**A**) Fluorescence intensity images. The fluorescence signal was acquired from 416 nm to 474 nm for the blue channel and from 474 nm to 532 nm for the green channel. (**B**) Generalized polarization (GP) images. The GP image was generated according to the GP function which is a normalized ratio between the blue and the green channels (GP scale from −1 to +1). According to the calculated GP values, we restricted the GP scale from −0.3 to 0.5. (**C**) GP histograms. The GP histogram was fitted using two Gaussian distributions. The lower GP values are associated with internal membranes, and the higher GP values are associated with plasma membranes. (**D**) Pseudo-colored GP images. The lower and higher GP distributions were pseudo-colored in green and red, respectively. (**E**) Averaged GP values. The GP values of individual cell lines were averaged and plotted; for each cell lines, a total of 12–18 images were used for statistical analysis. Significant differences were observed between mock control and IFITM1 (p = 0.00672), IFITM3 (p = 0.00107) in the plasma membrane. See text for details.

In principle, IFITM proteins could alter spontaneous monolayer curvatures and thereby restrict the creation of hemifusion by influencing the membrane molecular order and membrane fluidity. To explore these possibilities, we labeled 293/LH2SN cells expressing IFITM1, 2 or 3 or mock control with Laurdan, a hydrophobic fluorescent probe that is highly sensitive to lipid phases, and measured their generalized polarization (GP) values and florescence lifetimes using 2-photon laser scanning and fluorescence-lifetime imaging microscopy (FLIM) [52], [53]. Because of its large excited state dipole moment to align surrounding water molecule in the energy dissipation process, Laurdan has been commonly used to report the extent of water penetration into the lipid bilayer, which correlates with lipid packing [52], [54]. In general, higher GP values and longer lifetimes indicate that the membranes are more molecularly ordered, while lower GP values and shorter lifetimes mark membranes as less molecularly ordered [52]. In mock control cells, the GP distribution was characterized by two peaks, one with a lower GP, associated with intracellular membranes, and another with a higher GP, identified with plasma membranes; the less ordered populations were predominant in the mock controls (Figs. 8A–D; first row). In cells treated with methyl-β-cyclodextrin (MβCD), a cholesterol-depleting reagent known to make the membrane less ordered [55], we observed dramatically reduced GP signals, resulting in an almost complete loss of the higher GP peak in the histogram (Figs. 8A–D; second row). In sharp contrast, cells expressing IFITM1, 2 or 3 all exhibited marked increases in the GP value, which was particularly evident in the higher GP peak (Fig. 8A–D; third, fourth and fifth rows), suggesting that these cell membranes are more ordered than those of mock controls.

Quantitative analysis showed that the averaged GPs values of IFITM1 and 3 in the plasma membranes were significantly different from that of mock controls (p = 0.00672 and p = 0.00107, respectively) (Fig. 8E). The averaged GP values for the intracellular membranes of IFITM1 and 3-epxressing cells were also increased, albeit not statistically significant from those of mock controls (p = 0.08∼0.37) (Fig. 8E). Unfortunately, we have been unable to distinguish the lipid order of endosomal membranes from that of total intracellular membranes in this analysis. Noticeably, IFITM2 exhibited modest increases in the GP value (p = 0.1388) (Fig. 8E), which correlated its less inhibitory effect on syncytia formation and cell-cell fusion (Figs. 3, 4 and 5). The increased lipid order in cells expressing IFITM proteins was also evidenced by their longer FLIM lifetimes as compared to those of mock controls and MβCD-treated cells (Fig. S6). Collectively, these results showed that overexpression of IFITM proteins dramatically increases the lipid packing order of cell membranes and makes them less fluid and possibly less competent for membrane fusion (see below the Discussion).

## Discussion

The IFITM protein family is the first and thus far only restriction factor known to block viral entry [12], [56]. Previous studies have suggested that these proteins, particularly IFITM3, predominantly restrict viruses that fuse in the late endosomal or lysosomal compartments at a lower pH [12], [13], [14], [17], [18]. Here we provide evidence that this family of proteins can also effectively restrict viruses that fuse with a higher pH threshold, such as JSRV (∼pH 6.3) which requires both receptor binding and low pH to co-trigger membrane fusion activation. This activation process likely occurs in the GPI-anchored-protein-enriched endosomal compartment or caveolin-associated compartments [27], [29], [33]. Interestingly, we found that, among the three human IFITM proteins examined, IFITM1 was more active than IFITM2 and 3 in restricting JSRV Entry (Figs. 1A and B). This was not because of downregulation of JSRV Env or its Hyal2 receptor, nor due to a perturbation of receptor-mediated priming for fusion activation (Fig. 2). Instead, results of syncytia formation and cell-cell fusion experiments showed that JSRV Env-mediated fusion at low pH was profoundly inhibited by IFITM1 (Figs. 3, 4 and 5). Given that an extracellular low pH pulse cannot bypass the IFITM1-mediated inhibition of endosomal entry of JSRV (Fig. 4F), and that IFITM proteins do not inhibit 10A1 MLV Env-mediated fusion at neutral pH (Fig. 3), we conclude that the syncytia formation and cell-cell fusion assays employed in this study can reflect the situation of viral membrane fusion in endosomes, which is believed to be the predominant site of IFITM-mediated inhibition of viral entry.

Somewhat surprisingly, we observed that IFITM1 was also generally more effective than IFITM2 and 3 in suppressing syncytia formation induced by IAV HA (Fig. 3A) and VSV-G (not shown), even though these proteins restrict viral entry with almost comparable efficiency (Figs. 1A and B). The exact mechanism underlying these observations is currently unknown, but could be related, in part, to the relatively higher levels of IFITM1 expression on the cell surface in 293 cells as compared to that of IFITM2 and 3 (Fig. 1E and F). Quantitative cell-cell fusion analysis confirmed the syncytia formation data (Fig. 5), but also revealed that IFITM3 dramatically inhibited viral membrane fusion when COS7, rather than 293T, was used as effector cells (Figs. 4, 5 and Fig. S5). These results indicate that the effects of IFITM proteins on viral membrane fusion can be cell type dependent, which agrees with previous observations on viral entry [13], [15]. Because all three human IFITM proteins tested exhibited potent restriction of viral membrane fusion induced by all three classes of viral fusion proteins that have different structures (Fig. 5), we suggest that a common physical mechanism, rather than specific interactions with viral fusion proteins, is responsible. Results from our series of experiments support this hypothesis.

Our first line of evidence is the effect of CPZ on cell-cell fusion (Fig. 6). It has been previously established that CAS is a state of hemifusion, and that the addition of CPZ to cells at the CAS intermediate leads to full fusion [47], [57]. It is also known that for cells brought to CAS by fusion proteins that are triggered by acidic conditions, raising the temperature at neutral pH leads to full fusion [47], [48], [58]. We observed that the extent of aqueous dye spread in target cells expressing IFITM proteins was much less upon CPZ addition or upon raising the temperature from CAS than was fusion induced by lowering pH at 37°C (Fig. 6). These findings provide strong evidence that IFITM proteins inhibit the creation of hemifusion. Because the effects caused by adding CPZ or raising the temperature were qualitatively similar for JSRV Env and IAV HA (Figs. 6C and D), we conclude that the mechanism of inhibition by IFITM proteins is not dependent on the precise fusion protein. Although IFITM proteins may affect pore formation and/or expansion, their primary mechanism appears to be the prevention of hemifusion.

The second line of evidence for the IFITM-mediated block on hemifusion came from the OA experiments (Fig. 7). It has been repeatedly shown that hemifusion is promoted by negative spontaneous curvature and is inhibited by positive spontaneous curvature [51], [57]. Consequently, if IFITM proteins conferred positive spontaneous curvature to membranes that contain them, these proteins would naturally block hemifusion. As OA has a large negative spontaneous curvature [57], we reasoned that it should overcome the inhibitory actions of IFITM proteins if the curvature was at the core of the action of IFITMs. Experimentally, we observed that the addition of OA virtually overcame all of the block of fusion by IFITM proteins (Figs. 7A and B). That is, when hemifusion was induced by the addition of OA, pore formation readily resulted without any inhibition despite the expression of IFITM proteins in the target membrane. The fact that the addition of OA after establishing CAS had no apparent effect on IFITM-mediated inhibition further supports the conclusion that the block occurs at steps prior to the creation of hemifusion (Figs. 7A and B).

How can IFITM proteins block hemifusion? Our Laurdan labeling experiments showed that IFITM-expressing cell membranes were more ordered than those of mock controls, as evidenced by their increased GP values and longer FLIM lifetimes (Figs. 5, 8, and S6). The increase in the lipid order of IFITM-expressing cells, particularly in their plasma membranes, correlates with the potency of IFITMs in suppressing viral membrane fusion (Fig. 8 and Fig. S6). Thus, IFITM proteins may block hemifusion by decreasing the fluidity of the membrane that contains them: a decreased fluidity would reduce the ability of lipids to undergo movements necessary for achieving hemifusion. It is also possible that the increased exclusion of water from the bilayer, as indicated by the higher GP values in the presence of the proteins (Fig. 8) is due to an increased average area occupied by lipid headgroups relative to the area swept out by their acyl chains. This would be equivalent to a greater positive spontaneous curvature. It remains possible that expression of IFITM proteins alters the lipid composition of cell membranes, thereby influencing their fluidities and spontaneous curvatures. We emphasize that while changes in lipid order and membrane fluidity likely account for the general inhibitory effect of IFITMs on viral membrane fusion, they do not fully explain the virus-specific and somewhat cell type-dependent inhibition of IFITMs on viral entry as reported in this and previous studies (Figs. 1, 4 and 5; Fig. S5) [12], [13], [15]. Additional factors are likely to be involved, such as specific IFITM-binding partners and possibly viral elements that modulate IFITM-mediated inhibition of viral entry. In this respect, IFITM proteins may or may not influence cell-cell fusion mediated by developmental and cellular fusogens, depending on the specific cell systems that express IFITMs and cellular fusogens.

The reason IFITM proteins promote greater lipid order remains unclear, but we offer a suggestion. IFITM proteins may directly change membrane curvature by adopting an unconventional membrane topology or topologies that function as a wedge to generate positive spontaneous curvature. There is an increased appreciation that lipid-binding proteins, along with lipids themselves, can influence membrane curvature, which has been shown to be crucial for vesicular trafficking and membrane fusion [59], [60]. Results of continuum membrane mechanics show that the spontaneous curvature of the monolayer of the target membrane proximal to the membrane expressing the fusion proteins (i.e., outer leaflets) affects hemifusion, but the spontaneous curvature of the distal monolayer (i.e., inner leaflets) does not [61]. Given our experimental data showing that IFITM proteins block hemifusion (Figs. 6 and 7), we suggest that IFITM proteins affect the outer monolayer, probably by spanning part or all of the outer monolayer. This suggestion is in line with the predicted membrane topology of IFITM proteins [11], in which N- and C-termini face the lumens of vesicles. It is also supported by prior and our current work showing that both the N and C-terminally tagged epitopes of IFITM3 can be, though not prominently, detected by flow cytometry and immunostaining without permeabilization (Figs. 2E and F) [12], [14].

A recent study suggested that the mouse IFITM3 protein adopt an alternate topology [20]. In this report, the authors provided evidence that the originally predicted transmembrane domains of IFITM3 fold into a hairpin loop and span only the inner leaflets, resulting in an intramembrane topology with both N- and C-termini facing the cytosol [20]. While our data does not unambiguously demonstrate the existence of this alternate topology, it is possible that IFITM proteins adopts multiple and dynamic topologies. In fact, it has been shown that some transmembrane proteins, including those of viral glycoproteins, adopt dual or dynamic topologies because of “lipid flip-flop” and/or changes in the net charge of their cytosolic sequences [62], [63], [64]. Dynamic topologies could result from the cleavages of IFITM proteins that have been observed at both the N- and C-termini in mammalian cells [11], [19], [65] (our unpublished data). It is therefore possible that IFITM proteins, including their orthologs in different species which differ significantly at the N- and C-termini [11], adopt distinct topologies in mammalian cells; these sequence and topologic differences may account for, and contribute to, their somewhat distinct phenotypes in suppressing viral membrane fusion and entry into host cells.

## Materials and Methods

### Cells

293T, 293, HTX (a subclone of HT1080), COS7, 293T/GFP (stably expressing GFP), HAB2 (expressing IAV HA, kind gift of Judy White, University of Virginia, Charlottesville, VA), 293/LH2SN (stably expressing Hyal2), HTX/LH2SN (stably expressing Hyal2), and 293/GP-LAPSN (expressing MLV Gag-Pol and alkaline phosphatase (AP)) cells have all been described previously [27], [40], [46], [66]. COS7 cells expressing human Hyal2 were generated by transduction with PT67/LH2SN retroviral vector encoding human Hyal2 [40]. 293, HTX, 293/LH2SN, COS7/LH2SN, HTX/LH2SN cells stably expressing IFITM proteins were generated by transduction with pQCXIP (Clontech, Mountain View, CA) retroviral vectors encoding IFITM1, 2 or 3 (see below). K562 cells stably expressing control shRNA or shRNA targeting IFITM1 or 3 mRNA were kind gifts of Michael Farzan and I-Chueh Huang (Harvard Medical School, Boston, MA). All mammalian cells used were grown in Dulbecco's modified Eagle's (DMEM) medium with 10% FBS (Hyclone, Logan, UT).

### Plasmids and reagents

The human IFITM1, 2 and 3 genes, with or without an N-terminal FLAG tag, were amplified by PCR from pRetro-Tet-IFITM constructs [15]. PCR products were digested and ligated into the EcoRI/BamHI restriction sites of pQCXIP vector, resulting in pQCXIP-IFITMs. Retrovirus packaging plasmid encoding the MoMLV Gag-Pol (pCMV-gag-pol-MLV) and transfer vector encoding the GFP (pCMV-GFP-MLV) were kind gifts of Francois-Loic Cosset (INSERM U758-ENS, Lyon, France). Plasmids encoding JSRV Env with both N- and C-terminal FLAGs, the 10A1 amphotropic MLV Env, the vesicular stomatitis virus G protein (VSV-G) and SFV E1/E2 have been described previously [27], [29], [67], [68]. The 10A1 MLV Env construct with the R peptide deleted was created by removing the last 16 amino acid of the R peptide using PCR. Plasmids encoding the IAV HA and NA (Thailand KAN-1/2004 H5N1 strain) were kind gifts of Gary Nabel (NIH, Bethesda, MD). The MLV Gag-YFP construct was a kind gift of Walter Mothes (Yale University, New Heaven, CT). The anti-FLAG monoclonal antibody beads (EZview™-red), anti-FLAG antibody, anti-β-actin monoclonal antibody, anti-Tubulin, secondary anti-mouse immunoglobulin G conjugated to FITC, TRITC or HRP, chlorpromazine (CPZ), bafilomycin A1 (BafA1), and oleic acid (OA) were all purchased from Sigma (St. Louis, MO). Anti-IFITM1, anti-IFITM2 and IFITM3 were purchased from Proteintech Group (Chicago, IL). IFNα-2b, CMAC (7-Amino-4-Chloromethylcoumarin), calcein-AM, Methyl-β-cyclodextrin (MβCD), CMTMR (5-(and-6)-(((4-Chloromethyl)Benzoyl)Amino)Tetramethylrhodamine), and Lipofectamine 2000 were purchased from Invitrogen (Carlsbad, CA). The Express ^35^S-Met/Cys protein labeling mix was purchased from Perkin Elmer (Boston, MA). The JSRV SU-human IgG Fc fusion protein and sHyal2 were produced and purified as previously described [27], [41].

### Pseudovirion production, transduction and infection

The GFP- and AP-expressing MLV pseudovrions bearing JSRV Env, 10A1 MLV Env, IAV HA/NA and VSV-G were produced as previously described [33]. Target cells were infected with appropriate amounts of virus stock in the presence of 5 µg/ml Polybrene (Sigma), and assessed for GFP expression by flow cytometry 48 h after infection or for AP activity by staining of cells 72 h after infection. To test the effect of interferon (IFN) on viral entry, 293 cells were treated with 200–1000 units of IFN-α2b or medium alone for 24 h before pseudovirus infection. Typically, an MOI of 0.05 to 0.2 was used for all infections.

To create cell lines stably expressing IFITM1, 2 or 3, we produced retroviral pseudotypes by transfecting 293T cells with plasmids encoding IFITMs (pQCXIP-IFITMs), MLV-Gag-Pol (pCMV-gag-pol-MLV) and VSV-G (pMD.G) using the calcium phosphate method. Supernatants were harvested 48–72 h post-transfection and centrifuged at 3,200 g to remove cell debris. Cells were infected with pseudovirions in the presence of 5 µg/ml Polybrene. Twenty-four hour after infection, cells were selected in growth medium containing 1 µg/ml puromycin (Sigma). For production of MLV Gag-YFP pseudovirions bearing JSRV Env, 293/GP-LAPSN cells were co-transfected with plasmids encoding Gag-YFP and JSRV Env by the calcium phosphate method.

### Virus binding assay

MLV pseudovirions bearing JSRV Env and Gag-YFP were concentrated by centrifugation at 185,000 g on a 2 ml 20% sucrose cushion for 3 h, and were resuspended in phosphate-buffered saline (PBS). Cells were detached with PBS plus 5 mM EDTA, and incubated with different amounts of purified pseudovirions on ice for 3 h. The cells were washed with PBS for 5 times and fixed with 3.7% paraformaldehyde before being analyzed by using flow cytometry.

### Cell surface staining

Cells were detached by PBS containing 5 mM EDTA and resuspended in PBS plus 2% FBS. To examine the binding of JSRV SU to cells expressing Hyal2, 5×10^5^ HTX cells were incubated with 10 µg purified JSRV SU-human IgG Fc proteins on ice for 3 h, washed 3 times, and incubated with FITC conjugated anti-human IgG Fc antibody for another 1 h. Cells were then washed, fixed and analyzed by flow cytometry. For detection of the expression of the FLAG-tagged JSRV Env and IFITMs, similar procedures were used except that cells were incubated with an anti-FLAG antibody on ice for 1 h, followed by incubation with FITC conjugated anti-mouse IgG antibody for 1 h before cells were analyzed by flow cytometry.

### Metabolic labeling

Metabolic labeling was performed as previously described [27]. Briefly, 293T cells were transiently transfected with plasmids encoding JSRV Env and/or IFITMs by the calcium phosphate method. Twenty-four hours post-transfection, cells were starved in DMEM without cysteine and methinonine (MP Biomedicals, Cost Mesa, CA) for 30 min and pulse-labeled with a 62.5 µCi mixture of cysteine plus methionine (Perkin Elmer, Waltham, WA) for 1 h, followed by chase-labeling in complete growth medium. To examine shedding of JSRV SU, 3 h after the chase period, indicated amounts of sHyal2 were added and cells were incubated for another 3 h. Cell lysates and culture media containing the ^35^S-labeled JSRV Env were harvested and immunoprecipitated with anti-FLAG beads. Samples were then resolved by sodium dodecyl sulfate (SDS)-polyacrylamide gel electrophoresis (PAGE) and applied to autoradiography. Band intensities were quantified using Quantity One (Bio-Rad, Hercules, CA).

### Syncytium induction assay

Syncytia induction assays were performed as described previously [27]. Briefly, 293/LH2SN or COS7/LH2SN cells, either parental (mock) or derivatives stably expressing IFITMs, were seeded in 6-well plates and cotransfected with 2 µg plasmids encoding JSRV Env or 10A1 MLV Env with the R peptide deleted (10A1 Env-R^−^), or 0.5 µg plasmids encoding IAV HA, plus 0.5 µg peGFP-N1 (Clontech) using the calcium phosphate method. Twenty-four hours post-transfection, cells were treated with pre-warmed buffer (pH 6.2, pH 5.5, pH 5.0 etc.) for 1 min and were examined for syncytium formation under a fluorescence microscope. If applicable, cells were incubated with indicated doses of IFN-α2b for 24 h before being treated with a low pH buffer to induce syncytia. For 10A1 Env-R^−^, IFN-α2b was added upon transfection and was maintained throughout the entire fusion assay. The fusion index was calculated by using *f* = [1-(C/N)], where C is the number of cells per phase field after fusion, and N is the total number of nuclei [69]. At least five phase-contrast microscopy fields were used for the analysis, with means and standard deviations calculated.

### Cell-cell fusion assay

Two cell-cell fusion assays were employed in this study. The first cell-cells fusion assay was used for JSRV Env as previously described [27]. In brief, 293T/GFP cells were transfected with 2 µg plasmids encoding JSRV Env alone or plus IFITM1 by Lipofectamine 2000. Twenty-four hours after transfection, cells were detached with PBS plus 5 mM EDTA and co-cultured with CMTMR prelabeled effector HTX/LH2SN cell lines, either parental (mock) or derivatives expressing IFITM proteins. After co-culture for 1 h, cells were treated with a pH 5.0 buffer for 1 min, and recovered in complete growth medium for another 1 h. Cells were analyzed for fusion under a fluorescence microscope or by flow cytometry.

For a second cell-cell fusion assay, the viral fusion proteins (JSRV Env, VSV G, and SFV E1/E2) were separately expressed in COS7 cells to generate effector cells. In a few experiments, 293T cells were employed as effector cells to test whether the type of effector cell was of functional consequence. For IAV, the cell line HAB2 stably expressing influenza virus HA was used as the effector. 293/LH2SN cells stably expressing IFITM proteins, as described above, were the target cells. Effector cells were loaded with the fluorescent dye calcein-AM (Invitrogen) and targets were labeled by the dye CMAC (Invitrogen). For fusion experiments, cells were allowed to bind for 30 min at room temperature and pH was then lowered, through exchange of aqueous solutions, for 10 min, to 5.0 for JSRV Env, 4.8 for IAV HA, 5.7 for VSV G, and 5.4 for SFV E1/E2. The pH was then reneutralized to 7.2, and 30 min later fusion between pairs of effector and target cells was scored by the transfer of both aqueous dyes, as observed by fluorescence microscopy.

### Low pH rescue of BafA1 or IFITM1-mediated block on vial entry

Experiments were performed as previously described [33], [34]. Briefly, HTX or HTX/IFITM1 cells were pretreated with 20 nM BafA1 for 2 h and spininoculated with JSRV or IAV pseudovirions at 4°C for 1 h. Following three washes with cold PBS, the virion-cell complexes were either directly exposed to a pH 5.0 solution (for IAV) 5–10 min or were preincubated at 37°C for 1 h in the presence of 20 nM BafA1 (for JSRV) and then incubated with a pH 5.0 for 5–10 min. In both cases, the total period of infection in the presence of 20 nM BafA1 was 4 h. Noninternalized virus was inactivated using citrate buffer (pH 3.0) after the infection period, and viral infectivity was determined by counting AP-positive foci 72 h after the initiation of infection. A pH 7.5-PBS buffer and 0.01% DMSO served as controls for the pH 5.0 buffer and 20 nm BafA1, respectively.

### Creating and using CAS

After binding labeled effector and target cells on cover slips within culture dishes at room temperature for 30 min, the dishes were placed on ice, bringing the solutions bathing the cells to 4°C. The pH was lowered (∼pH 5.0) for 10 min before reneutralizing at 4°C to pH 7.2. At this point, the cells were in a “cold-arrested” stage (CAS). After 3 min, one of two operations was performed. In the first, 0.5 mM CPZ was added (through exchange of solutions at 4°C) and 1 min later the CPZ was washed out with a solution containing delipidated-BSA. Each dish was kept on ice, and each cover slip was removed to monitor aqueous dye transfer by fluorescence microscopy. In the second manipulation, cells at CAS were placed in a 37°C incubator for 30 min and dye transfer was then monitored. In order to make spontaneous monolayer curvatures more negative, 285 µM oleic acid (OA, Sigma) was incorporated into cell membranes either prior to or subsequent to creating CAS. For prior incorporations, effector and target cells were incubated together at room temperature for 20 min and OA was then added; 15 min later, the solutions were lowered to 4°C and CAS was created. OA was removed at 4°C by washing the cells with a solution containing delipidated-BSA. CPZ was then added or temperature was raised to 37°C. To incorporate OA subsequent to CAS, OA was added to cells at CAS, and CPZ was added or the temperature was raised without removing OA.

### Laurdan labeling

The membrane probe Laurdan (6-dodecanoyl-2-dimethylamino naphthalene, Invitrogen) was dissolved in DMSO (dimethylsulfoxide) to make a stock concentration of 1.8 mM. 293/LH2SN cells expressing IFITM1, 2 or 3, or none (Mock) were incubated with 1.8 µM Laurdan for 40 min at 37°C. To deplete cholesterol, a 50 mM stock solution of methyl-β-cyclodextrin (MβCD, Sigma-Aldrich) was prepared by dissolving in nanopure water. Cells were incubated with 10 mM MβCD for 1 h at 37°C. All cells were rinsed with PBS once before being processed for imaging.

### Microscope handling, imaging, and data analysis involved in Laurdan labeling

In order to quantitatively assess the membrane order, a ratiometric method known as generalized polarization (GP) was developed [52]. The GP function or value characterizing the spectral properties of Laurdan is calculated through the following expression:
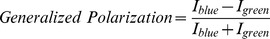
(1)where *I_blue_* and *I_green_* are the respective intensities conventionally centered at 440 nm (the emission maximum for more ordered lipid bilayer) and centered at 490 nm (the emission maximum for less ordered lipids).

GP and FLIM data were acquired with a Zeiss LSM710 META Laser scanning microscope, coupled to a 2-Photon Ti:Sapphire laser (Spectra-Physics Mai Tai, Newport Beach, CA) producing 80 fs pulses at a repetition of 80 MHz and a ISS A320 FastFLIMBox for the lifetime data. A 40× water immersion objective 1.2 N.A. (Zeiss, Oberkochen, Germany) was used for all experiments. The excitation wavelength was set at 780 nm. A SP 760 nm dichroic filter was used to separate the fluorescence signal from the laser light. For FLIM data, the fluorescence signal was directed through a 495 LP filter and the signal was split between two photo-multiplier detectors (H7422P-40, Hamamatsu, Japan), with the following bandwidth filters in front of each: blue channel 460/40 and green 540/25, respectively. For image acquisition, the pixel frame size was set to 256×256 and the pixel dwell time was 25.61 µs/pixel. The average laser power at the sample was maintained at the mW level. For GP data the fluorescence signal was acquired from 416 nm to 474 nm for the blue channel and from 474 nm to 532 nm for the green channel, using the spectral detector of the LSM 710 by joining 6 channels of the detector each having a bandwidth of 9.7 nm. For image acquisition, the pixel frame size was set to 256×256 and the pixel dwell time was 177.32 µs/pixel. The average laser power at the sample was maintained at the mW level. SimFCS software developed at the Laboratory for Fluorescence Dynamics (www.lfd.uci.edu) was used to acquire FLIM data and to process FLIM and GP data. Calibration of the system and phasor plot for FLIM data was performed by measuring fluorescein (pH 11), which has a known single exponential lifetime of ∼4.04 ns. Calibration for GP data was performed with Laurdan in DMSO which has a reference GP value equal to zero.

GP histograms of the images were calculated using SimFCS, and the histograms fitting was performed by using a MatLab routine. GP histograms were characterized by the presence of two Gaussian distributions. After highlighting the corresponding pixels in the images, it was possible to couple the first Gaussian distribution (centered at lower GP values) with the intracellular membranes of the cell, and the second distribution (centered at higher GP values) with the plasma membranes [70]. The two histogram distributions were used to calculate the averaged GP values of intracellular and plasma membranes.

### Statistical analysis

Paired student *t* test was used for statistical analysis unless otherwise noted. Typically data from three to eight independent experiments were used for the analysis.

## Supporting Information

Figure S1 **Effect of wildtype IFITMs on JSRV, 10A1 MLV, IAV and VSV entry.** Experiments were performed as described in Fig. 1 except that HTX cells expressing the wildtype IFITM1, 2 or 3 were used for infection.
(TIFF)

Figure S2 **Examination of the expression of IFITMs in 293 cells.** 293 or 293 cells stably expressing IFITM1, 2 or 3 were treated with IFN-α2b (500 units/ml) for 24 h or left untreated, and cell lysates were examined for IFITM expression by Western blot using anti-IFITM1, anti-IFITM2, anti-IFITM3, and anti-FLAG antibodies, respectively. Tubulin served as a loading control, which was determined by an anti-Tubulin antibody. Note that the levels of endogenous IFITM expression induced by IFN-α2b in 293 cells were much less than those of IFITM overexpression.
(TIFF)

Figure S3 **Effect of different pH on syncytia formation induced by JSRV Env.** Experiments were performed similarly as described in Fig. 3, except that indicated pH buffers were applied. Experiments were performed three times, with similar results obtained. Representative images are shown.
(TIFF)

Figure S4 **Effect of wildtype IFITMs on syncytia formation induced by JSRV Env and IAV HA.** Assays were carried out as described in Fig. 3, except 293/LH2SN cells expressing the wildtype IFITM1, 2 or 3 were used. Experiments were repeated at least 4 times, with similar results obtained. Representative images are presented.
(TIFF)

Figure S5 **Human IFITM3 inhibits JSRV Env-mediated entry and cell-cell fusion in COS7 cells.** (**A**) COS7/LH2SN cells expressing human IFITM1, 2 or 3 were infected with GFP-encoding MLV pseudovirions bearing JSRV Env or IAV HA/NA; 24 h after infection, infectious titers were determined by flow cytometry. Flow cytometry profiles from one typical experiment are shown. (**B**) Experiments were performed as described in Fig. 3, except that COS7/LH2SN cells expressing IFITM1, 2 or 3 were transfected with plasmid encoding JSRV Env or IAV HA, and that syncytia formation was examined following a pH 5.0 treatment for 5 min. For each cell line, the representatives of both phase-contrast and GFP images are shown; arrows indicate syncytia induced by JSRV Env.
(TIFF)

Figure S6 **Effect of IFITM expression on the lipid order of cell membranes examined by FLIM.** Cells were analyzed by fluorescence-lifetime imaging microscopy (FLIM). FLIM images were acquired by using ISS A320 FastFLIMBox. SimFCS software developed at the Laboratory for Fluorescence Dynamics (University of California, Irvine) was used to acquire FLIM data and to process FLIM and GP data. The Phasor approach was used to directly visualize the Laurdan lifetime distribution and to associate a color map to lifetime values (see reference 53). Note that green cursors are associated with shorter lifetimes or less ordered lipid membranes (e.g. MβCD-treated cells), while red cursors correspond to longer lifetimes, ordered lipid membranes (e.g., IFITM-expressing cells). (**A**) Fluorescence intensity image. (**B**) FLIM image in the green channel. (**C**) Phasor plot. (**D**) Phasor color palette distribution.
(TIFF)
