# Supplementary figures and images for: IFITM Proteins Restrict Viral Membrane Hemifusion
Source: PLoS Pathog. 2013 Jan 24;9(1):e1003124. doi: 10.1371/journal.ppat.1003124 (PMC3554583; doi:10.1371/journal.ppat.1003124)

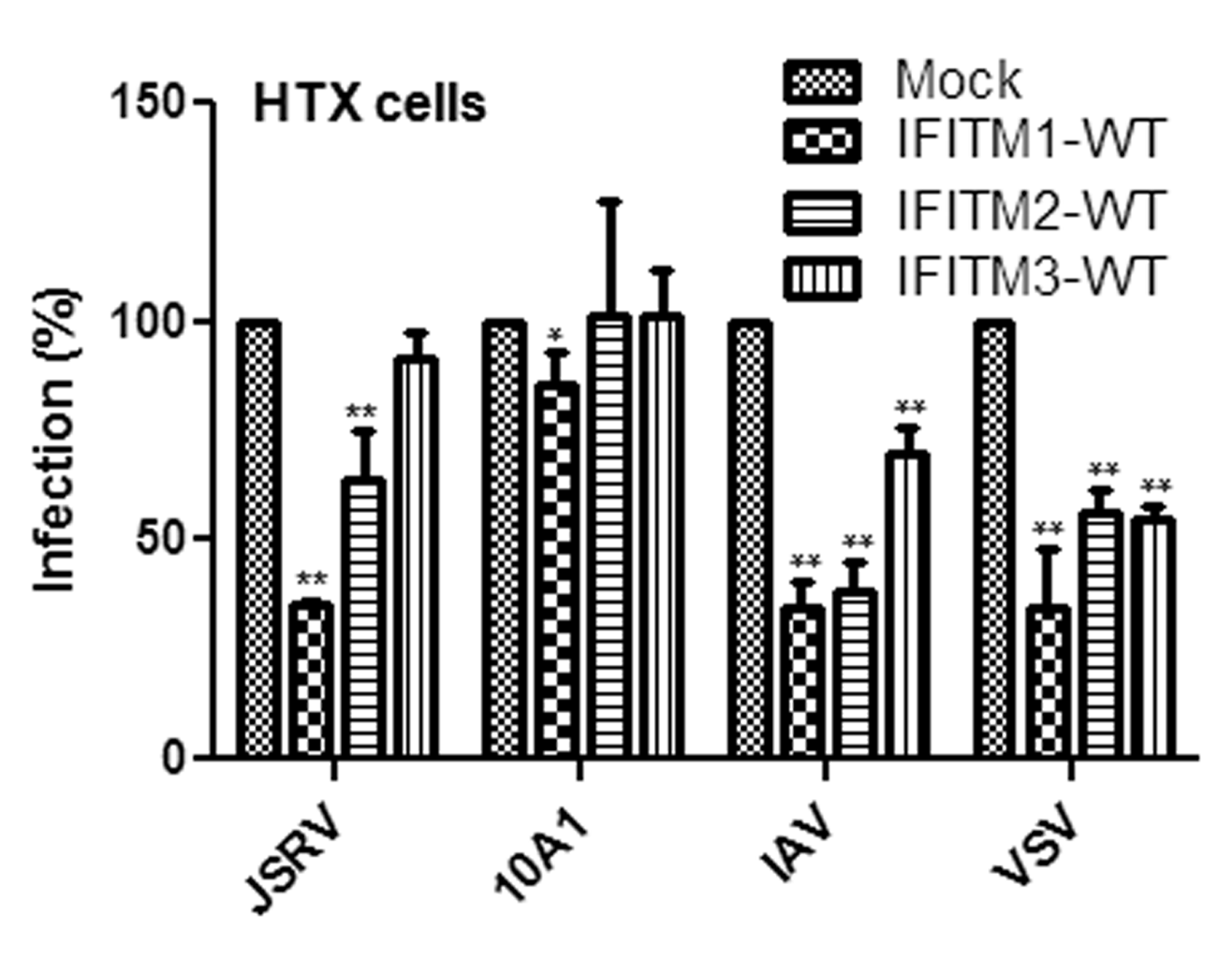

Supplement: Figure S1 — Effect of wildtype IFITMs on JSRV, 10A1 MLV, IAV and VSV entry. Experiments were performed as described in Fig. 1 except that HTX cells expressing the wildtype IFITM1, 2 or 3 were used for infection. (TIFF) [file ppat.1003124.s001.tiff]

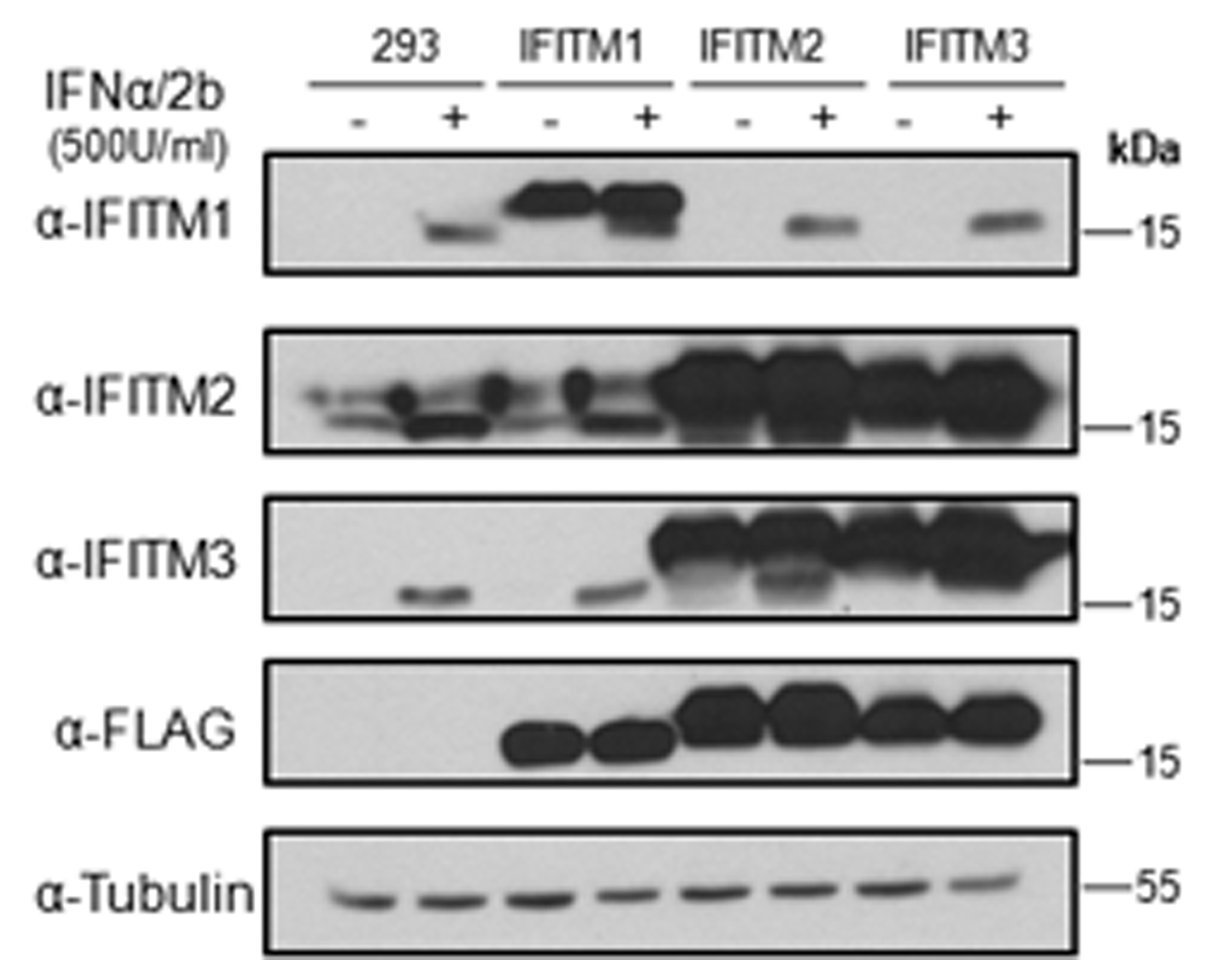

Supplement: Figure S2 — Examination of the expression of IFITMs in 293 cells. 293 or 293 cells stably expressing IFITM1, 2 or 3 were treated with IFN-α2b (500 units/ml) for 24 h or left untreated, and cell lysates were examined for IFITM expression by Western blot using anti-IFITM1, anti-IFITM2, anti-IFITM3, and anti-FLAG antibodies, respectively. Tubulin served as a loading control, which was determined by an anti-Tubulin antibody. Note that the levels of endogenous IFITM expression induced by IFN-α2b in 293 cells were much less than those of IFITM overexpression. (TIFF) [file ppat.1003124.s002.tiff]

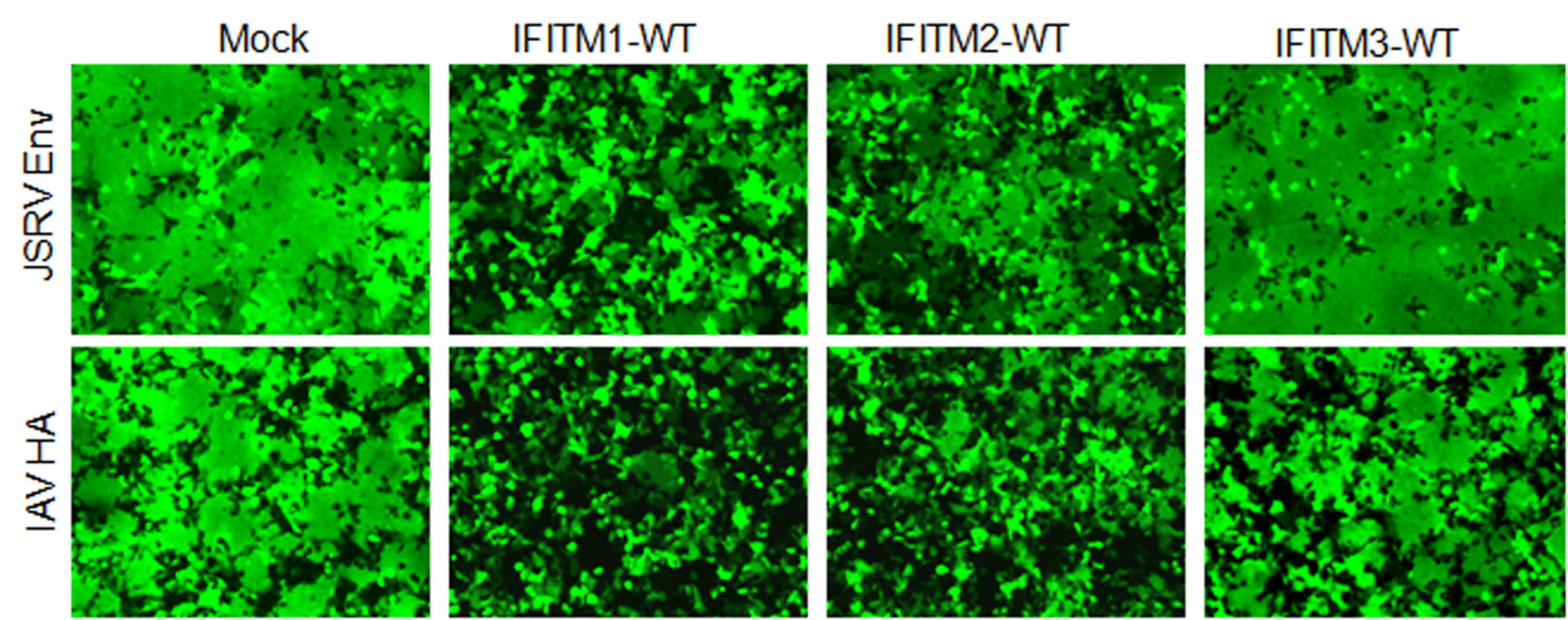

Supplement: Figure S3 — Effect of different pH on syncytia formation induced by JSRV Env. Experiments were performed similarly as described in Fig. 3, except that indicated pH buffers were applied. Experiments were performed three times, with similar results obtained. Representative images are shown. (TIFF) [file ppat.1003124.s003.tiff]

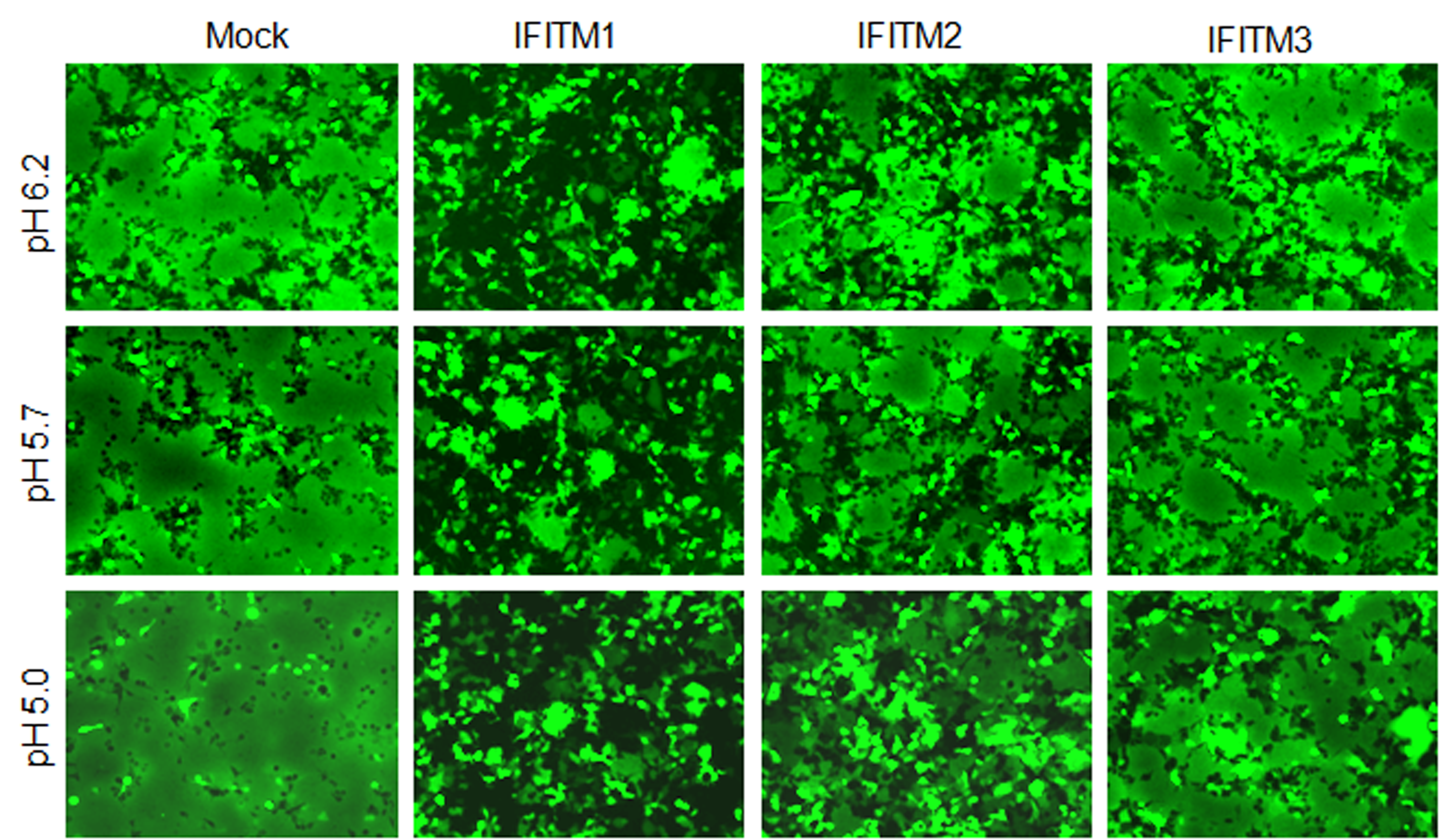

Supplement: Figure S4 — Effect of wildtype IFITMs on syncytia formation induced by JSRV Env and IAV HA. Assays were carried out as described in Fig. 3, except 293/LH2SN cells expressing the wildtype IFITM1, 2 or 3 were used. Experiments were repeated at least 4 times, with similar results obtained. Representative images are presented. (TIFF) [file ppat.1003124.s004.tiff]

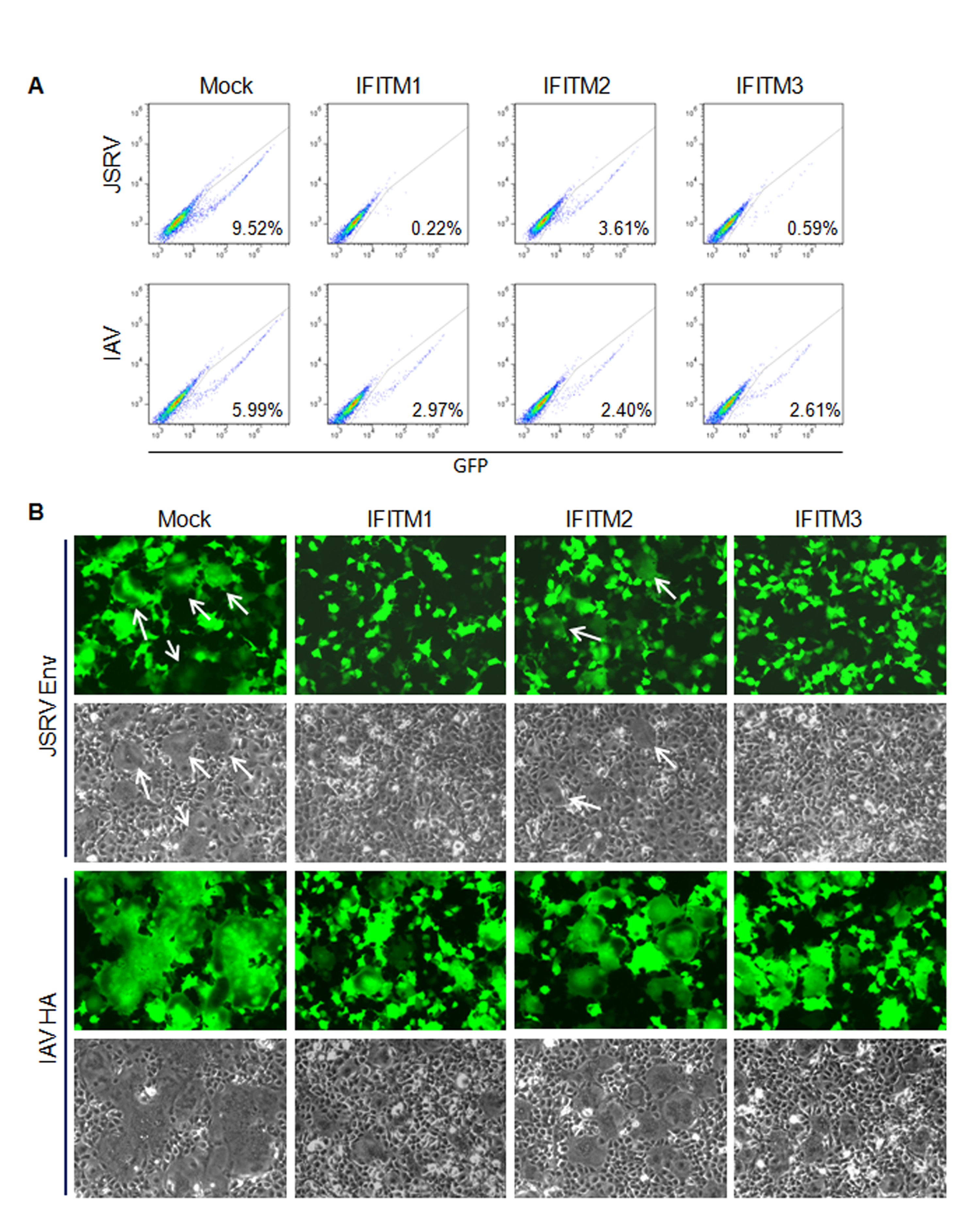

Supplement: Figure S5 — Human IFITM3 inhibits JSRV Env-mediated entry and cell-cell fusion in COS7 cells. (A) COS7/LH2SN cells expressing human IFITM1, 2 or 3 were infected with GFP-encoding MLV pseudovirions bearing JSRV Env or IAV HA/NA; 24 h after infection, infectious titers were determined by flow cytometry. Flow cytometry profiles from one typical experiment are shown. (B) Experiments were performed as described in Fig. 3, except that COS7/LH2SN cells expressing IFITM1, 2 or 3 were transfected with plasmid encoding JSRV Env or IAV HA, and that syncytia formation was examined following a pH 5.0 treatment for 5 min. For each cell line, the representatives of both phase-contrast and GFP images are shown; arrows indicate syncytia induced by JSRV Env. (TIFF) [file ppat.1003124.s005.tiff]

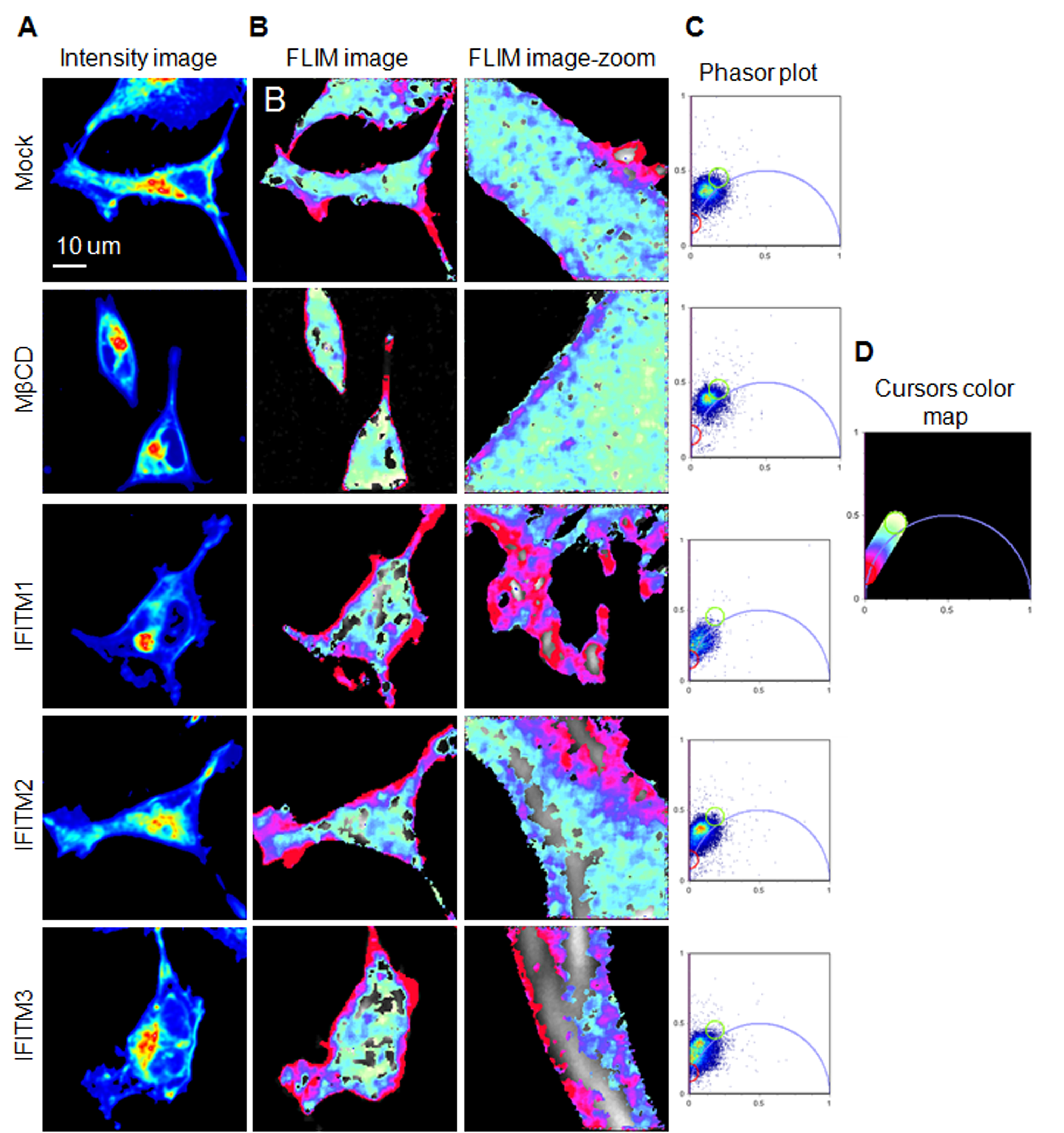

Supplement: Figure S6 — Effect of IFITM expression on the lipid order of cell membranes examined by FLIM. Cells were analyzed by fluorescence-lifetime imaging microscopy (FLIM). FLIM images were acquired by using ISS A320 FastFLIMBox. SimFCS software developed at the Laboratory for Fluorescence Dynamics (University of California, Irvine) was used to acquire FLIM data and to process FLIM and GP data. The Phasor approach was used to directly visualize the Laurdan lifetime distribution and to associate a color map to lifetime values (see reference 53). Note that green cursors are associated with shorter lifetimes or less ordered lipid membranes (e.g. MβCD-treated cells), while red cursors correspond to longer lifetimes, ordered lipid membranes (e.g., IFITM-expressing cells). (A) Fluorescence intensity image. (B) FLIM image in the green channel. (C) Phasor plot. (D) Phasor color palette distribution. (TIFF) [file ppat.1003124.s006.tiff]
